# Supplementary material for: Binning unassembled short reads based on k-mer abundance covariance using sparse coding
Source: Gigascience. 2020 Mar 29;9(4):giaa028. doi: 10.1093/gigascience/giaa028 (PMC7099633; doi:10.1093/gigascience/giaa028)
Supplement: giaa028_GIGA-D-19-00410_Original_Submission [file giaa028_giga-d-19-00410_original_submission.pdf]

GigaScience

Binning unassembled short reads based on k-mer covariance using sparse coding
--Manuscript Draft--

**Manuscript Number:**	GIGA-D-19-00410
**Full Title:**	Binning unassembled short reads based on k-mer covariance using sparse coding
**Article Type:**	Research
**Funding Information:**	Investissements d'Avenir (FSN-CISN2 ADAMme) — Not applicable
**Abstract:**	Background Sequence binning techniques enable the recovery of a growing number of genomes from complex microbial metagenomes and typically require prior metagenome assembly, incurring the computational cost and drawbacks of the latter, e.g. biases against low-abundance genomes and inability to conveniently assemble multi-terabyte datasets.<br><br>Results We present here a scalable pre-assembly binning scheme (i.e. operating on unassembled short reads) enabling latent genomes recovery by leveraging sparse dictionary learning and elastic-net regularization, and its use to recover hundreds of metagenome-assembled genomes, including very low-abundance genomes, from a joint analysis of microbiomes from the LifeLines-Deep population cohort (n=1135, > 1010 reads).<br><br>Conclusion We showed that sparse coding techniques can be leveraged to carry out read-level binning at large scale, and that despite lower genome reconstruction yields compared to assembly-based approaches, bin-first strategies can complement the more widely used assembly-first protocols by targeting distinct genome segregation profiles. Read enrichment levels across six orders of magnitude in relative abundance were observed, indicating that the method is able to recover genomes consistently segregating at low levels.
**Corresponding Author:**	Thomas Brüls<br>Commissariat a l'Energie Atomique et aux Energies Alternatives Centre de Saclay<br>FRANCE
**Corresponding Author Secondary Information:**	
**Corresponding Author's Institution:**	Commissariat a l'Energie Atomique et aux Energies Alternatives Centre de Saclay
**Corresponding Author's Secondary Institution:**	
**First Author:**	Olexiy Kyrgyzov
**First Author Secondary Information:**	
**Order of Authors:**	Olexiy Kyrgyzov
	Vincent Prost
	Stéphane Gazut
	Bruno Farcy
	Thomas Brüls
**Order of Authors Secondary Information:**	
**Additional Information:**	
**Question**	**Response**
Are you submitting this manuscript to a special series or article collection?	No

Powered by Editorial Manager® and ProduXion Manager® from Aries Systems Corporation

**Experimental design and statistics**<br><br>Full details of the experimental design and statistical methods used should be given in the Methods section, as detailed in our Minimum Standards Reporting Checklist. Information essential to interpreting the data presented should be made available in the figure legends.<br><br>Have you included all the information requested in your manuscript?	Yes
**Resources**<br><br>A description of all resources used, including antibodies, cell lines, animals and software tools, with enough information to allow them to be uniquely identified, should be included in the Methods section. Authors are strongly encouraged to cite Research Resource Identifiers (RRIDs) for antibodies, model organisms and tools, where possible.<br><br>Have you included the information requested as detailed in our Minimum Standards Reporting Checklist?	Yes
**Availability of data and materials**<br><br>All datasets and code on which the conclusions of the paper rely must be either included in your submission or deposited in publicly available repositories (where available and ethically appropriate), referencing such data using a unique identifier in the references and in the "Availability of Data and Materials" section of your manuscript.<br><br>Have you have met the above requirement as detailed in our Minimum Standards Reporting Checklist?	Yes

Powered by Editorial Manager® and ProduXion Manager® from Aries Systems Corporation

Manuscript Click here to download Manuscript Kyrgyzov.pdf

GigaScience, 2017, 1–12

doi: xx.xxxx/xxxx
Manuscript in Preparation
Paper

PAPER

Binning unassembled short reads based on k-mer covariance using sparse coding

Olexiy Kyrgyzov[1], Vincent Prost[1,2], Stéphane Gazut[2], Bruno Farcy[3] and Thomas Brüls[1,*]

[1]CEA Genoscope and [2]CEA LIST and [3]Bull Technologies

[*]bruls@genoscope.cns.fr

Abstract

Background Sequence binning techniques enable the recovery of a growing number of genomes from complex microbial metagenomes and typically require prior metagenome assembly, incurring the computational cost and drawbacks of the latter, e.g. biases against low-abundance genomes and inability to conveniently assemble multi-terabyte datasets.
Results We present here a scalable pre-assembly binning scheme (i.e. operating on unassembled short reads) enabling latent genomes recovery by leveraging sparse dictionary learning and elastic-net regularization, and its use to recover hundreds of metagenome-assembled genomes, including very low-abundance genomes, from a joint analysis of microbiomes from the LifeLines-Deep population cohort (n=1135, > 10^{10} reads).
Conclusion We showed that sparse coding techniques can be leveraged to carry out read-level binning at large scale, and that despite lower genome reconstruction yields compared to assembly-based approaches, bin-first strategies can complement the more widely used assembly-first protocols by targeting distinct genome segregation profiles. Read enrichment levels across six orders of magnitude in relative abundance were observed, indicating that the method is able to recover genomes consistently segregating at low levels.

Key words: Metagenomics; Human microbiome; Sequence binning; Sparse coding

Background

Metagenomic shotgun sequencing has dramatically increased our appreciation of the intricacies of microbial systems, whether sustaining biogeochemical processes or underlying health status of their hosts. Several limitations, including sequencing errors, strain-level polymorphism, repeat elements and inequal coverage, among others, concur however to yield fragmented metagenome assemblies, requiring post-processing in order to cluster (bin) assembled fragments into meaningful biological entities, ideally strain-resolved genomes.

The advent of reasonably efficient sequence binning techniques, often exploiting a coverage covariance signal across multiple samples, allowed the field of metagenomics to move toward more genome-centric analyses[1], and recently thousands of so-called metagenome-assembled genomes (MAGs) have been reported, both from environmental sources and human surfaces or cavities[2, 3, 4, 5]. The vast majority of these MAGs have been produced by post-assembly binning approaches, i.e. operating on sequence contigs assembled on a sample by sample basis. Though highly successful, such methods are nevertheless "inherently biased towards the most abundant organisms, meaning consistently less abundant organisms may still be missed" (quoted from ref[4]). For example, although thousands of MAGs were reconstructed from more than 1500 public metagenomes in the remarkable study [2], over 93% of these MAGs had an average coverage of more than 10x in at least one of the samples analyzed. The high proportions of phylogenetically unassigned reads typical in medium to high complexity metagenomes is another consequence of this limitation[6].

Even though the ecological or community-level importance of rare species is a matter of debate, there are both theoreti-

Compiled on: November 27, 2019.
Draft manuscript prepared by the author.

cal and empirical observations supporting the notion that rare organisms can substantially contribute to community-level behavior and resilience, hence represent valuable targets for genome recovery.

Theoretical modeling of microbial trade of diffusible goods[7] have for example highlighted an apparent paradox (called “curse of increased efficiency” by the authors of ref[7]), where one bacterial species becomes rarer in the population despite becoming fitter and more efficient at producing a key metabolic resource. This situation is provoked by metabolic interdependencies that can evolve via trade in microbial consortia, and that can lead to low-abundance organisms becoming essential for a faster growth rate of the community. On the other hand, several empirical studies have documented the ecosystem-level relevance of rare bacteria (see ref[8] for a review), for example ref[9] makes a case for the role of “ultrarare” bacteria in ecosystem-level productivity, and ref[10] highlight the role of some low abundance bacteria in driving termite’s hindgut bacterial community composition.

Considering that global metagenome assembly (or cross-assembly) is currently unpractical to recover low abundance genomes or complex microbial consortia from terabytes of data, we decided to investigate a “bin first and assemble second” paradigm that could make the assembly problem more tractable by targeting lower complexity sequence subsets (bins). Binning unassembled reads is however more computationally demanding, as the number of raw sequences is typically orders of magnitude higher than the number of assembled contig sequences.

Even though the dominating paradigm nowadays is assembly-first binning, it is worth noting that the first sequence binning methods reported, like AbundanceBin[11] and MetaCluster[12], operated at the read level. This shift towards contig binning was mainly driven by the increase in data throughput, as the first read-level binning methods were designed at the time of 454 and even Sanger sequencing (both providing longer reads) to process individual samples. They were thus not designed to scale to large multi-sample terabase-sized short read datasets. In this perspective, assembly can be viewed as a pre-processor to reduce the computational burden of binning.

A pioneering pre-assembly binning scheme[13] was proposed a couple of years ago, with the read partitioning problem formulated by analogy to the latent semantic analysis (LSA) technique widely used in natural langage processing (NLP). The core idea to view metagenomes as linear mixtures of genomic variables can lead to read clustering formulations based on the deconvolution of latent variables (“eigengenomes”) driving the k-mer (subsequences of length k) abundance covariance across samples. The raw sequence data is first summarized in a sample by k-mer occurrence matrix (analogous to term-document matrices in NLP), approximating the abundance of k-mers across samples. Matrix decomposition techniques can then be used to define two sets of orthogonal latent vectors analogous to principal components of sample and sequence space. The large memory requirements incurred by the factorisation of large abundance matrices naturally drove ref[13] toward a rank-reduced singular value decomposition (SVD), for which efficient streaming libraries[14] enable a parallel processing of blocks of the abundance matrix by updating the decomposition iteratively. Clusters of k-mers can then be recovered by an iterative sampling and merging heuristic that samples blocks of eigen k-mers from the right singular vectors matrix until an arbitrary portion (about 0.4% in ref[13]) of the latter has been covered. This heuristic is however acknowledged as a significant hindrance, the authors calling for “more sophisticated methods [are needed] to computationally discover a natural clustering” (quoted from ref[13]).

We describe here a pre-assembly binning method based on sparse dictionary learning and elastic-net regularization that exploits sparsity and non-negativity constraints inherent to k-mer count data. This sparse coding formulation of the binning problem can leverage efficient online matrix factorization techniques[15] and scales to very large (terabyte-sized) k-mer abundance matrices; it also bypasses the aforementioned problematic k-mer clustering heuristic, removes interpretability issues associated with the SVD (e.g. the physical meaning of negative contributions), and is able to enrich sequences from a given genome across 6 orders of magnitude in relative abundance (see section "Recovery of very low-abundance genomes" thereafter).

Analyses

We describe in the following section some analyses and results of the proposed binning scheme based on the modeling of data vectors as sparse linear combinations of basis elements (sparse coding [15]).

We will start with a preliminary experiment illustrating the ability of read binning to recover a target genome whose sequences segregate at levels too low to yield and kilobase-sized fragment by assembly in any single sample, hence would not be recoverable by assembly-first approaches. We will then describe results from a direct comparison of assembly-first versus bin-first methods that illustrate the complementarity of the two approaches in terms of the profiles of genomes recovered. The next subsection describes a comparison of the sparse coding based bin-first approach with a state of the art read-binning method. The next subsections will describe strain separation results obtained with the new method, document its scalable behavior, and its ability to enrich rare sequences, enabling the recovery of low abundance genomes. We will conclude with a discussion of some important limitations of the method and consider some of its potential applications.

Read-level binning can recover low abundance genomes that escape assembly-first protocols

We devised an experiment to illustrate a situation where assembly-first approaches are not able to recover a target genome -because target genome sequences are too low in number in any single sample- whereas a bin-first approach is successful at it. The experimental setup involved distributing a very low number of short reads (100 paired reads) randomly sampled from a target genome (a 10 kbp plasmid) into 14 samples containing each a background of 20000 unrelated bacterial sequences (4 further samples contained only background sequences with no read from the target genome at all). As no single kilobase-sized fragment could be recovered by assembling the sequences from each sample individually, this precluded the application of assembly-first methods (e.g. contig binning methods like metabat[16, 17] require >= 1500 bp sequences as input). On the other hand, circa 90% of the reads originating from the target genome could be segregated in a single cluster/bin using our read binning pipeline (Supplementary Table 1), leading to the complete recovery of the target genome in a single contig after assembly (Methods).

Bin-first and assembly-first strategies recover distinct and complementary genome sets

A second experiment aimed at directly comparing the genome recovery yield of assembly-first versus bin-first strategies on a real-life dataset. We selected the raw sequence data from 18

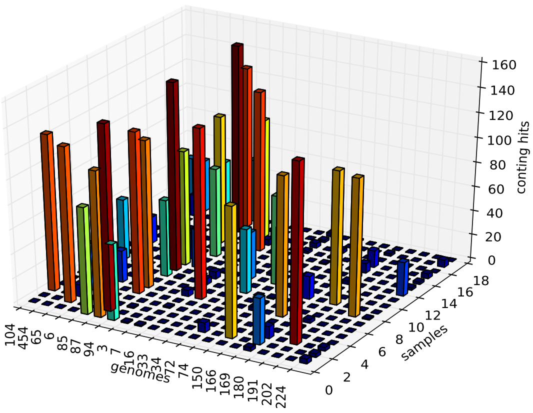

Figure 1. Sample origin of the sequences aggregated into genome bins (displayed by their genome identifier on the x-axis) using our bin-first method (first seven genomes on the left) versus assembly-first binning using metabat2 (14 rightmost genomes). Genomes retrieved by the bin-first method aggregate sequences from a larger number of samples.

(randomly chosen) individuals of the LifeLinesDeep cohort[18], and either assembled these individually (i.e. on a sample by sample basis) with metaSPAdes (v3.13.0) followed by contig binning across samples with the MetaBat2 adaptive algorithm [17], or clustered the raw reads using our read-level binning pipeline, followed by metaSPAdes assembly of the resulting partitions/bins.

Fourteen nearly (>90%) complete and uncontaminated (<5%) genomes were recovered using the assembly-first approach, versus 7 using the bin-first method. Interestingly, the two genome sets were disjoint, with no complete genome recovered by both approaches. Among the 14 genomes recovered by the assembly-first approach, three were not represented in the set of 164 MAGs recovered from the analysis of the entire cohort. More surprisingly, only 3 out of the 7 complete genomes retrieved by our bin-first pipeline from the analysis of 18 samples were represented among the complete or nearly complete MAGs identified from the full cohort analysis, indicative of a lack of stability of the algorithm that we relate to bin fragmentation provoked by extensive strain-level variation across the samples (see Discussion).

The surprising lack of overlap between the two genome sets in this experiment is not attributable to fundamental differences in abundance levels between the genomes recovered by the two approaches, as in both cases the genome bins could be directly aligned to individual sample assemblies, i.e. the genomes recovered using both approaches were of sufficiently high coverage to yield relatively large contigs in the assemblies of individual samples. We assessed potential differences between the distributions of binned genome sequences across the samples, which highlighted distinct patterns for the two approaches, with the genomes identified by the bin-first approach aggregating sequences from a larger number of samples (and harboring a higher number of contigs per genome bin on average) (Figure 1).

Thus, in the present experiment, the assembly-first approach targeted genomes reaching high abundance in a limited number of samples, for which the weaker abundance covariation signal probably hampered the bin-first approach. Consistent with this view, sequences from genomes produced through the assembly-first approach were frequently located in large (dozens of Mbp in size) and unresolved partitions computed by read-level binning.

On the other hand, we should keep in mind that the number of samples (18) used in this experiment is relatively

	K-means	LSA	Sparse Coding
Precision	0,52	0,58	0,72
Recall	0,63	0,64	0,82
F-Value	0,57	0,61	0,77

Table 1. Binning accuracy estimates: LSA refers to the original algorithm of ref[13], with a cosine similarity threshold of 0.7 as recommanded by the authors, k-means refers to a direct clustering of the columns of the abundance matrix, with the number of clusters set to 1000 (equal to the number of components for the sparse decomposition), see main text and Methods.

low. Related approaches based on abundance-covariance, like Concoct[19] or LSA[13] among others, require a higher number of samples to achieve best performance (about 50 samples for the former and between 30 to 50 for the latter).

Despite these limitations, the fact that the bin-first approach was able to recover a significant number of complete genomes not identified by the assembly-first approach illustrates the complementarity of the two strategies.

Enhanced accuracy of sparse coding based read binning versus state of the art read binning

Besides the two pioneering read binning methods already mentioned (AbundanceBin[11] and MetaCluster[12]), we could also mention CompostBin[20], which is a PCA-based read level binning algorithm that was designed and tested on Sanger reads. BiMeta[21] and MetaProb[22] are other tools that operate at the read-level, but describe themselves as “assembly-assisted”, meaning they rely on the detection of read overlaps. BiMeta was tested on 454 reads simulating bacterial communities of a dozen of different genomes at most and on the Acid Mine Drainage dataset of ref[23], which is of low complexity and consists in Sanger reads. MetaProb shares some principles with BiMeta: it is also “assembly-assisted” and was tested on the same low-complexity synthetic datasets as the latter. The authors also tested their method on a real microbiome sample consisting in 43 million reads, but only after filtering the latter down to 2 million reads.

Thus, all the above methods were designed to operate on a individual samples, at a time were scalability issues were less acute. Moreover, with the exception of AbundanceBin which exploits a coverage signal extracted from unique k-mers, the other methods are better described as composition-based, using a nucleotide composition signal measured from short k-mers (typically of length 4 or 5).

We developed our method with scalability in mind, as we wanted it to be able to process on the order of 10^{10} short reads and to be able to process increasingly larger multi-sample datasets by simply stacking additional computing resource. In this respect, there is only one competing method left, Latent Strain Analysis[13], that is both scalable and designed to operate on unassembled short reads from multiple samples.

To evaluate our method, we first compared its read clustering accuracy (measured in terms of precision, recall and F-value metrics, see Methods) with that of the original LSA method by using previously described benchmark datasets[24] (downloadable from http://www.genoscope.cns.fr/SCdata/vc50/), for which read to genome assignments were known (ref[24] and Methods). The results from these experiments are summarized in Table 1, and show improved accuracy of the sparse-coding framework over both the original LSA and a naive k-means algorithms.

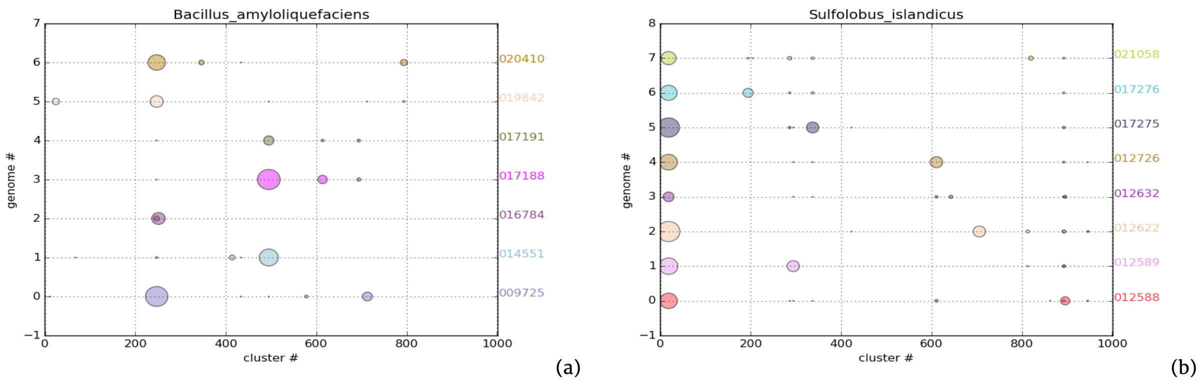

Figure 2. Partial resolution of species pangenomes. x-axis: partition identifier, y-axis: horizontal axes correspond to different strains from the same species (left: *B. amyloliquefaciens* strains, right: *S. islandicus* strains). Circle area is proportional to the number of reads from a given strain assigned to the given partition. The left panel illustrates the partial separation of seven strains in two distinct partitions. The right panel illustrates the differential segregation of the core (at the left of the figure) and variable portions of the species pangenome.

1						
94.20	1					
94.30	99.44	1				
94.21	99.96	99.44	1			
98.74	94.20	94.27	94.20	1		
99.02	94.25	94.33	94.25	98.72	1	
97.75	94.09	94.20	94.09	97.79	97.77	1

Table 2. Average Nucleotide Identity (ANI) between the B. amyloliquefaciens strains used in the strain separation experiment illustrated in the left panel of Figure 2 (see main text and Methods)

Partial strain separation

The counting and indexing of k-mers in fixed memory is achieved by locality sensitive hashing (Methods). By design, locality sensitive hash functions increase the probability of collision for related items[25]. On one hand, this provides a convenient way to handle sequencing errors. On the other hand, the occurrence in natural environments of multiple strains from the same species (the so-called species pangenome) could lead to artefactual k-mer merging and potential overlap between distinct genomic partitions. This represents an issue potentially exacerbated by the inter-sample read aggregation process.

To assess the behavior of the method in the presence of extensive pangenomic (i.e. strain-level) variation, we quantified its ability to separate closely related (up to 99.96% average nucleotide identity (ANI), Table 2) strains that were deliberately included in the genome mixtures from the virtual cohort used in the test experiments.

The two panels of the Figure 2 illustrate two practical examples of partial strain separation achieved with the method. The left panel illustrates a partial separation of 7 strains from the *Bacillus amyloliquefaciens* species (whose ANI ranged from 94.18 to 99.96, Table 2), while the right panel shows similar results for 8 strains of the *Sulfolobus islandicus* species (whose ANI ranged from 97.84 to 99.59). As the genomic origin of each read is known in the virtual cohort dataset, these plots show, for each strain (represented by a horizontal line), the distribution of its reads among the full set of clusters/bins generated by the pipeline (and arbitrarily ordered along the x-axis). The left panel illustrates that the 7 strains from the B. amyloliquefaciens species are mostly separated into two groups according to whether their main cluster is located near x-coordinate 220 or x-coordinate 500. The right panel on the other hand shows that the 8 strains of the S. islandicus species share a common "core" cluster (located near the origin), while variable portion of their genomes are segregated into distinct "variable" clusters.

Overall, this analysis makes apparent a partial separation of closely related strains (left panel), as well as the differential segregation of the core (i.e. the genome fraction that is shared between all the strains of a species) and variable portions of the species pangenomes (right panel).

In practice, some level of strain mix-up is probably inherent to the inter-sample read aggregation process, and approaches based on sample by sample assembly limit the risk of strain mixing, but at the expense of focusing on those genomes that reach high-coverage (around 10x). Our approach aimed at relaxing the latter constraint, but by doing so through the aggregation of lower abundance reads across samples, it becomes vulnerable to extensive strain-level variation. Dealing with this problem is the focus of future research, e.g. a possible workaround could be to carry out a "soft-clustering" by allowing "core" sequences to belong to more than one "variable" cluster.

Sensitivity and scalability on real-life data

By scalability, we refer to the ability of the method to adapt to order of magnitude change in the input (raw reads), and its ability to maintain its functionality and performance under high demand (i.e increasingly higher data volumes).

To assess the sensitivity and scalability of the sparse coding method, we applied it to a real world dataset of over 10^{10} reads (about 10 terabytes of raw sequence data) derived from 1135 gut microbiomes of healthy Dutch individuals from the LifeLinesDeep cohort[18]. The pre-assembly binning of the cohort's reads resulted in 983 partitions, which were then assembled individually using the Spades engine[26] (Methods). The distribution of assembly sizes is shown in Figure 3, making apparent that the vast majority of partitions are bacterial-genome sized (i.e. in the 2-5 Mbp range). A few dozens of coarse-grained partitions harboring unresolved genomes makes up the right tail of the distribution. As a direct read to genome mapping is not available for real life metagenomes, we assessed clustering performance by quantifying the genomic homogeneity and completeness of the resulting partitions based on the occurrence pattern of universal single-copy markers using the checkm toolkit[27]. A summary of completion and contamination statistics of the genome-resolved partitions is presented in Table 3, while another facet of the homogeneity of recon-

Classification	Completeness	Genomes (Bins)	Contamination
Nearly Complete	>90%	14	≤ 5%
Substantial	>70% to ≤ 90%	53	≤ 5%
Moderate	> 50% to ≤70%	97	≤ 5%
Partial	≤ 50%	724	≤ 5%
Unresolved	>100%	95	> 5%

Table 3. Genome completion and contamination statistics of assembled partitions/bins, see main text and Methods.

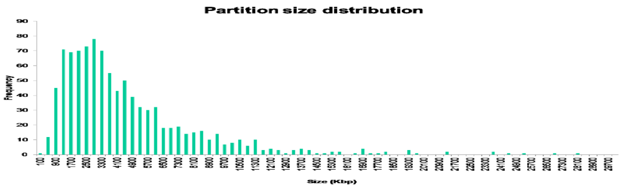

Figure 3. Distribution of assembled bin sizes. x-axis: assembled partition size (in kbp), y-axis: partition frequency

structed genomes is displayed in the left panels of Figure 5.

The fact that many of the partitions display low contamination is somehow balanced by the concomitant generation of large and unresolved partitions. The production of these unresolved partitions arises from the fact that the extent of genome divergence is not uniform across the range of taxa occurring in the samples. As discussed above, strain-level ("pangenomic") variation is another factor contributing to cluster fragmentation, by inducing a differential segregation of the core and variable portions of genomes, and is exacerbated by the inter-sample read aggregation process.

Recovery of very low-abundance genomes

A key motivation for the pre-assembly processing of reads was the theoretical possibility to aggregate reads from low abundance organisms across samples.

To assess whether we could indeed identify such consistently low abundance genomes in real-life datasets, we characterized the abundance of a subset of > 70% complete genomes from the LifeLinesDeep cohort analysis by directly mapping the raw reads of the original samples against them. Given the large size of the cohort, this analysis was not performed on the full set of MAGs but on a limited number of genomes, the aim being to validate the ability of the method to retrieve such low-abundance genomes by exhibiting some of them.

The relative enrichment levels of these genomes was measured as the fraction of raw reads contributed by each sample to them (Methods), and is illustrated in Figure 4 for two genomes, with the left panel showing an example of a consistently low-abundance genome (i.e. with nearly all the samples contributing no more than 10^{-5} to 10^{-4} of their reads to the given genome), while the right panel shows a genome of overall moderate abundance (10^{-4}) but reaching higher abundance (10^{-3}) in a few dozens of samples (represented by the rightmost peak in the histogram).

Given the large number of microbiomes analyzed, we quite frequently observed situations where a given genome reaches medium to high relative abundance in at least one sample (as illustrated in the right panel of Figure 4). However and importantly, we could also detect instances of genomes that consistently segregated at low abundance levels across the whole cohort (left panel of Figure 4 and right panels of Figure 5).

The recovery of these genomes was made possible by aggregating a few thousands reads per sample across a large number of samples, thus demonstrating the ability of the method to isolate rarer genomes. Overall, the high proportion of homogeneous partitions corresponding to partial genomes (Table 3) is consistent with the recovery of sequences from lower abundance organisms, whose cumulative coverage across the cohort is not sufficient to allow complete genome reconstruction.

Assessing novelty against reference genome compendia

To investigate the extent to which the recovered genomes could correspond to novel organisms, we screened a subset of 164 of them (more than 50% complete with less than 5% contamination, accessible at http://www.genoscope.cns.fr/SCdata/MAGs/) against several reference genome libraries. We first compared the genomes against the Kraken 2[28] database built from NCBI's Refseq bacteria, archaea and viral libraries (on October 2018). Only 21 out of the 164 genomes compared had at least one fragment classified against this reference database (Methods). We also compared the genomes against the "Global Human Gastrointestinal Bacteria Genome Collection" (HGG, ref[6]), that represents one of the most comprehensive resources of gastrointestinal bacterial reference sequences currently available. Only less than half (72/164) of the genomes displayed convincing similarity to the HGG genome catalogue (Methods).

Discussion

Covariance-based binning has the power to identify biologically meaningful associations between metagenomic sequences that could go unnoticed by analyses based on sequence overlap (assembly) or nucleotide signatures. This is illustrated in the present study by a preliminary experiment using a synthetic dataset spiked with low abundance sequences from a target genome that does not reach a sufficient coverage to yield kilobase-sized fragments after assembly in any individual sample (thus precluding the application of contig binning), but which is successfully recovered via read-level binning (Supp. Table 1). When applied to the > 10^{10} reads from the LifeLinesDeep cohort's metagenomes, our bin-first protocol recovers hundreds of metagenome-derived genomes, including from consistently less abundant organisms (Figure 4 and right panels of Figure 5). By increasing the number of distinct abundance profiles that can be generated, larger sample numbers increase both the sensitivity and resolution of covariance-based methods; one may therefore anticipate further gains in the application of such methods in relation to future increases in the scale of sequence data generated (e.g. increased cohort sizes).

We need however to acknowledge several important limitations that impede the overall performance and applicability of our bin-first framework.

First, we already mentioned a limitation arising from the natural occurrence of strain-level variation at the origin of differential segregation of core and variable fractions of species pangenomes (Figure 2). The large number of incomplete but otherwise uncontaminated partitions/bins in the LifeLlinesDeep analysis partly reflects the widespread occurrence of this type of variation in natural habitats. However, it should be noted that assembly-based approaches are neither immune to this type of variation, frequently discarding it when building "flattened" consensus contigs. This type of polymorphism is difficult to handle in a *de novo* way, and current methods for strain-level surveys of metagenomes typically rely on reference databases of strain-specific nucleotide polymorphisms

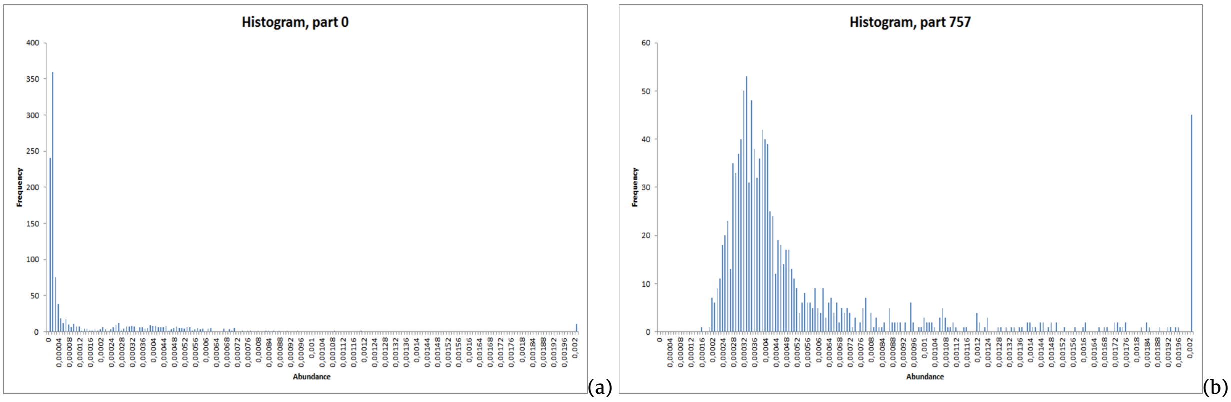

Figure 4. Enrichment histograms displaying the fraction of raw reads contributed by each sample to two distinct genome-resolved bins. x-axis: read abundance of partition 0 (left) and partition 757 (right); y-axis: sample frequency (among the 1135 samples). Different situations are illustrated: a relatively high proportion of reads can be contributed by a small subset of individuals (a few dozens, corresponding to the rightmostest peak for the genome-resolved bin shown in panel b), while panel a illustrates that substantial (i.e. $\geq$70% complete) genomes of low-abundance organisms can also be recovered by aggregating only a few thousands reads per sample across the full cohort.

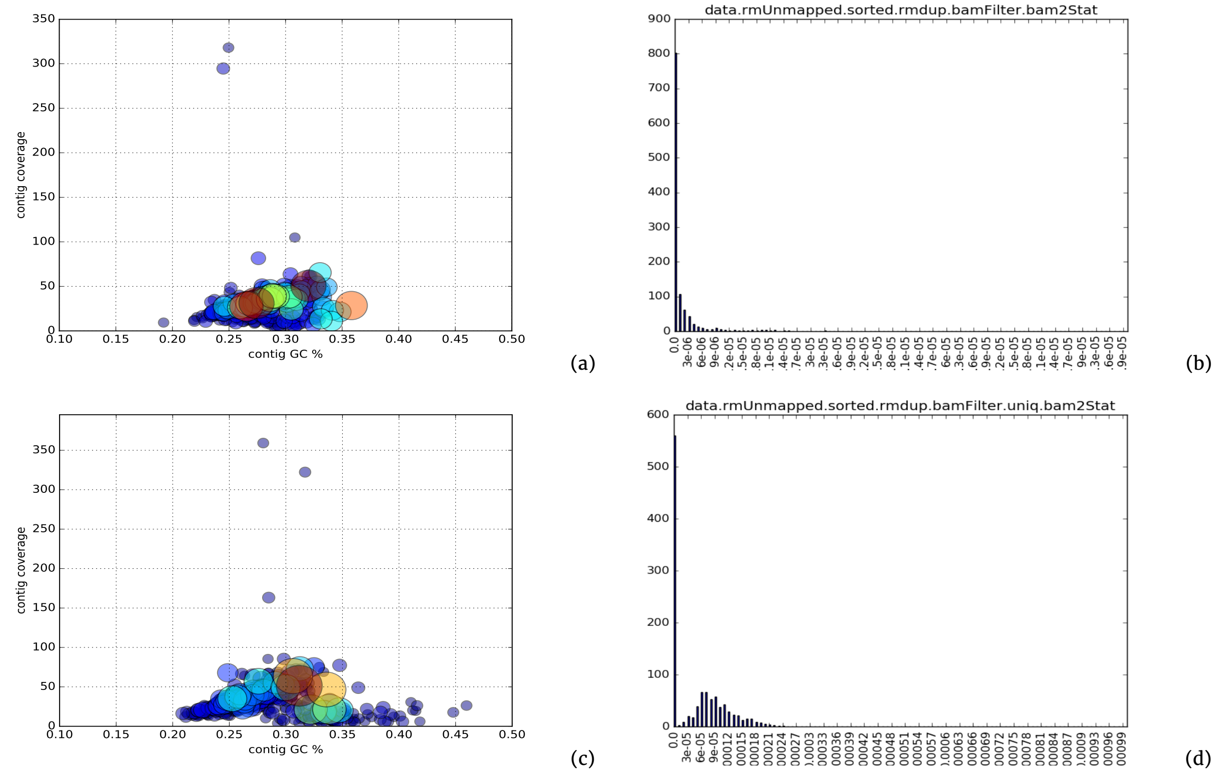

Figure 5. Left panels (a,c): GC-coverage plots (x-axis: contig GC%; y-axis: contig coverage) illustrating the homogeneity of two assembled bins (top: bin 470 (70%complete, 4.8% contamination) bottom: bin 766 (70% complete, 3.5% contamination)) corresponding to two unclassified Firmicutes genomes of low-abundance, whose enrichment histograms are shown in the corresponding right panel (b,d). Right panels (b,d): Enrichment histograms showing the fraction of raw reads contributed by each of the 1135 samples to the two genomes whose GC-coverage plots are displayed in the corresponding left panel. x-axis: read abundance of genome bins 470 (panel b) and 766 (panel d); y-axis: sample frequency (among the 1135 samples).

(see for example ref[29]). Sample by sample assembly limits the risk of strain mix-up, but at the expense of focusing on those genomes that reach high-coverage (around 10x). Our approach aimed at relaxing the latter constraint, but by doing so through the aggregation of lower abundance reads across samples, it becomes vulnerable to extensive strain-level variation.

To the best of our knowledge, a method that could target -in an unsupervised way- low-coverage genomes in a strain resolved manner is not available today, and working towards this goal is a promising research area. It should be noted however that, to some extent, the degree of similarity that one wishes to distinguish can be tuned through the choice of the k-mer length. Increasing the latter would increase the separation of closely related sequences, but only to some extent because the locality sensitive hashing scheme will by nature increase the probability of collision for similar sequences. Thus, here again we face another benefit *vs* disadvantage trade-off: besides efficient in-memory indexing, LSH allows to conveniently handle sequencing errors (noise), but this can also put a limit on the power to separate very similar sequences (e.g. strains).

The observation that 4 out of 7 genomes retrieved in the preliminary experiment based on 18 samples were not among the set of MAGs identified by analyzing the full dataset is indicative of a lack of stability of the algorithm. This effect of the sample number is most likely mediated by the increasing presence of strain variation when aggregating reads across increasing numbers of samples, leading to more fragmented partitions. We probably underestimated the extent of strain-level variation in real-world data, and the high-level of genome fragmentation in the LifeLinesDeep partitions can be partly attributed to this problem, with low sequence coverage obviously contributing as well.

Another limitation of the method is the generation of coarse-grained partitions harboring a large number of unresolved genomes (corresponding to the tail of the partition size distribution shown in Figure 3). This problem is already manifest in the preliminary experiment comparing assembly-first versus bin-first approaches, and further exemplified in the large cohort analysis that yielded 983 partitions, 888 of which displayed low-levels (<5%) of contamination (Table 3), but also produced several large clusters holding dozens of microbial genomes. The generation of such unresolved partitions appears difficult to avoid as the extent to which genomes differ to each other is variable across phylogenetic groups. As a result, there is no single setting (e.g. k-mer length, hash size) that could achieve perfect separation of genomes from highly diverse genome mixtures.

These two limitations probably concur to explain that the number of moderate to nearly complete genomes recovered from the population cohort analysis appears much lower than the number of "species genomes" recoverable via assembly-first approaches (remember for example that close to 5,000 species-level genome bins were recovered from the analysis of nearly 10,000 metagenomes in ref[3] (one should however note that an average of 5.3 Gb per sample *after* quality control was generated in the latter study, versus 3.0 Gb *before* quality control in the LLDeep[18] data used here).

When analyzing a large number of related samples, we noticed quite commonly that distinct organisms are able to reach a sufficiently high (to be assembled) relative abundance level in at least one sample (a situation exemplified in the right panel of Figure 4). When following a sample by sample assembly-based strategy, a high coverage reached in a single sample (the likelihood of which increases with the number of samples analyzed) might be sufficient to assemble significant portions of a genome, even if it segregates at much lower levels in the remaining part of the cohort. This probably contributes to explain the high genome recovery yields of assembly-based approaches.

However, a key feature of the presented method is its ability to recover genomes of organisms consistently segregating at low levels across the entire cohort, as verified in a test experiment and on real-world data (cf left panel of Figure 4). The observation that more than half of the genomes recovered here were not detected in a very large compendium of human gut genomes assembled from thousands of samples[3] is consistent with this view.

Metagenomic sequence binning is still a very active research field, and there are many interesting ongoing efforts, including some attempts to cast binning as an assembly-graph partitioning problem[30]. Recent efforts include ref[31] that exploits structural sparsity of compact De Bruijn assembly graphs to compute succinct indexes in linear time, allowing to perform neighborhood queries on large assembly graphs in an "assembly-free" manner. Nevertheless, most of the recent development efforts in the field of metagenomic sequence binning remain directed toward assembly-first approaches, which have already delivered a vast array of performent and user-friendly software[32, 19, 33, 34], some of which have shown capabilities to recover genomes as low as 0.6 % (10^{-3})in relative abundance[33]. However, we have shown that the method presented here is able to recover genomes by sequence enrichments of the order of up to 10^{-6} (10^{-7} for some plasmid sequences), and therefore believe that it could be a useful adjunct to existing more mainstream approaches, especially for targeting more rare organisms.

Potential implications

As global metagenome assembly (and even more coassembly) remains unpractical for multi terabase-sized datasets, methods like the one described here -for which computer memory requirements remain independent of sequence depth- could prove valuable by making pre-assembly binning tractable while allowing to gain access to genomes from the rare biosphere.

Methods

Control datasets

The dataset described in ref[24] (and accessible from http://www.genoscope.cns.fr/SCdata/vc50/) corresponding to a virtual cohort of 50 individuals each harboring a microbiome of 100 distinct bacterial genomes sampled under a power-law abundance distribution (with power parameter α = 1.0) from a pool of 750 fully-sequenced genomes at an average depth of 10x (see ref[24] for details), was used in the control experiments. We call these datasets semi-synthetic because they are made of real bacterial genome sequences assembled into artificial mixtures. The read to genome assignments (ground truth) being known in advance for all the reads, the precision and recall metrics were computed from the read clustering output as in equations (10) and (11) from ref[35] (see section "Comparison of read binning algorithms"), with precision corresponding to what the authors refer to as purity and recall corresponding to completeness.

Real dataset: LifeLines-DEEP metagenomes

The LifeLines-DEEP cohort features 1135 individuals (474 males and 661 females) from the general Dutch population, whose gut microbiomes were shotgun sequenced using the Illumina short read technology, generating an average of 32 mil-

lion reads per sample, see [18] and EBI dataset accession number EGAD00001001991.

Locality Sensitive Hashing (LSH)

We used the SimHash[25] scheme described in ref[13] to obtain a proxy for k-mer abundance. Briefly, raw reads are parsed into k-mers of fixed size (k=31 was used in our experiments), the bases of which are individually mapped to a complex simplex via a mapping of the form: A=1, C=i, G=-i, T=-1, that can also incorporate base-call confidence scores[13]. k-mers are thus represented in k-dimensional space in which n hyperplanes (we used $n = 30$ in our experiments) are randomly drawn, creating 2^n subspaces, or buckets, indexing the columns of the sample by k-mer abundance matrix, whose rows were scaled to unit ℓ_2 norm. The LSH scheme is sequence sensitive, increasing the probability of collision for more similar k-mers[25], and allows the representation of k-mer abundance matrices of arbitrary dimensions in fixed memory.

Regarding the choice of a k-mer length, the key requirement is that k-mers should be sufficiently long so that most of them will be specific to each genome, thereby capturing genuine abundance patterns of individual genomes. In our experiments, the k-mer length (31) was chosen to be close to the value used by the authors of ref[13] to analyze their largest (terabase-sized) dataset. Some limited experiments with varying k-mer length values were carried out on smaller subsets of the data to check that small variations in k-mer size did not result in disproportionate differences in clustering outputs.

In choosing the k-mer length, we were also guided by the observations in ref[36] that k-mer similarity between genomes at different k approximates various degrees of taxonomic similarity, with k=31 appearing to correspond to species-level similarity. We also noticed that k=31 is the default setting in the popular sequence classification engine kraken[28].

Sparse coding

Our aim is to learn sparse and non negative factors from the sample by (hashed) k-mer abundance matrix $\mathbf{X}$. The sparsity assumption has biological roots in the fact that every individual only harbors a small subset of all the genomes that constitute the global microbiome, while each genome only contains a very small subset of the k-mers encountered across all the samples. Sparse coding aims at modeling data vectors as sparse linear combinations of elements of a basis set (aka dictionary) that can be learned from the data by solving an optimization problem[15]. We used the spams library (http://spams-devel.gforge.inria.fr/), that implements the learning algorithm of ref[15]: given a training set $\mathbf{x}^1, \ldots, \mathbf{x}^n$ it tries to solve

$$\min_{\mathbf{D}\in C} \lim_{n\to+\infty} \frac{1}{n} \sum_{i=1}^{n} \min_{\alpha_i} \left(\frac{1}{2} \parallel \mathbf{x_i} - \mathbf{D}\alpha_\mathbf{i} \parallel_2^2 + \psi(\alpha_\mathbf{i}) \right)$$

where ψ is a sparsity-inducing regularizer (e.g. the ℓ_1 norm) and C is a constraint set for the dictionary (positivity constraints can be added to α as well). The following optimization scheme was used (FL stands for fused lasso):

$$\min_{\mathbf{D}\in C} \frac{1}{n} \sum_{i=1}^{n} (1/2) \parallel \mathbf{x_i} - \mathbf{D}\alpha_\mathbf{i} \parallel_2^2 + \lambda_\mathbf{1} \parallel \alpha_\mathbf{i} \parallel_\mathbf{1} + \lambda_\mathbf{2} \parallel \alpha_\mathbf{i} \parallel_2^2$$

with C a convex set verifying

$$C = \mathbf{D} \in \mathbf{R}^{\mathbf{m}\times\mathbf{p}} \mathbf{s.t.} \forall \mathbf{j}, ||\mathbf{d_j}||_2^2 + \gamma_\mathbf{1}||\mathbf{d_j}||_\mathbf{1} + \gamma_\mathbf{2}\mathbf{FL}(\mathbf{d_j}) <= \mathbf{1}$$

Once the dictionary has been learned, the spams library offers an efficient implementation of the LARS algorithm[37] for solving the Lasso or Elastic-Net problem: given the data matrix $\mathbf{X}$ in $\mathbf{R}^{m\times n}$ and a dictionary $\mathbf{D}$ in $\mathbf{R}^{m\times p}$, this algorithm returns a matrix of coefficients $\mathbf{A} = [\alpha^\mathbf{1}, \ldots, \alpha^\mathbf{n}]$ in $\mathbf{R}^{p\times n}$ such that for every column $\mathbf{x}$ of $\mathbf{X}$, the corresponding column α of $\mathbf{A}$ is the solution of

$$\min_{\alpha}(1/2)||\mathbf{x} - \mathbf{D}\alpha||_2^2 + \lambda_\mathbf{1}||\alpha||_\mathbf{1} + (\mathbf{1/2})\lambda_\mathbf{2}||\alpha||_2^2$$

The spams implementation of this algorithm allows to add positivity constraints on the solutions α, which have a natural interpretation as weighing the contribution of the different hashed k-mers to the latent genomes. In practice, we defined clusters by assigning hashed k-mers from bucket i to component c if $c = \mathrm{argmax}_j A_{i,j}$.

Read classification and Assembly

Starting with the raw reads and their decomposition into k-mers, the bulk of the binning algorithm thus operates in k-mer space. After computing co-varying k-mers sets ("eigengenomes"), a post-processing step is thus necessary to assign reads to their cognate k-mer clusters in order to achieve a read-level clustering. We sticked to the LSA procedure[13] for this step, with the original reads being assigned to k-mer clusters based on a log-likelihood score aggregating i) cluster sizes (measured in terms of k-mer numbers), ii) the overlap between k-mers in reads and those in clusters, iii) an inverse document frequency (IDF)-style weight expressing the rarity of each of the overlapping k-mers. After read assignment, the partitions were assembled with the Spades (v3.13.0) engine[26] using default settings.

First experiment for comparing assembly-first versus bin-first protocols

An experimental setup was designed to illustrate the ability of read binning to cluster rare reads form a target genome across samples, while assembly-first protocols are inoperable because the low coverage of the target genome prevents the generation of any kilobase-sized contig through the assembly of the individual samples.

The dataset consisted in 18 samples each containing a subset of 20000 reads sampled from the 18 metagenomic libraries analyzed in ref[38] and randomly spiked with mock reads from a Bacillus thuringiensis plasmid (NG_035027.1) as in the test data used in the original LSA paper[13]. However, as the number of spiked reads (up to 4000) distributed among the samples in LSA's test dataset was sufficient to yield contigs covering a large fraction of the plasmid genome upon assembly, we derived a new dataset only containing 0 to 100 paired-reads (14 samples contained 100 paired-reads while 4 were entirely devoid of plasmid reads). This dataset is available on the website associated to this publication's material.

After checking that no kilobase-sized contig could be assembled in any of the samples -thus precluding the application of contig binning- the dataset was processed by our pre-assembly pipeline using the following settings: a k-mer length of 30 and a hash size of 22 were used to build the k-mer abundance matrix, the latter was decomposed by a svd and the

columns of the eigen-kmer matrix were clustered using a cosine similarity threshold of 0.25, followed by read assignment and assembly (using Spades) of the partitions. More than 99% (2782 out of 2800) of the plasmid derived reads ended up in a single partition (Supp. Table 1, leading to the recovery of the complete target genome upon assembly.

Second experiment for comparing assembly-first versus bin-first strategies

The raw sequence data from 18 (randomly chosen) individuals of the LLDeep cohort were either assembled individually (i.e. on a sample by sample basis) with metaSPAdes (v3.13.0) followed by contig binning across samples with the MetaBat2 adaptive algorithm [17], or the raw reads were clustered using our read-level binning pipeline, followed by metaSPAdes assembly of the resulting partitions/bins.

The raw reads were first mapped to the assembled contigs using bwa-mem[39] using default parameters. MetaBat2 was then invoked in the following way: first, the jgi_summarize_bam_contig_depths script was called to compute contig abundance statistics from the read mapping bam files, with the default options (minimum percent identity for a mapped read: 0.97, minimum contig length: 1000, minimum contig depth: 1). The metabat2 program was then called using the default parameters (minCV 1.0, minCVSum 1.0, maxP 95%, minS 60, and maxEdges 200) and the previously generated coverage statistics file, leading to the generation of 225 bins covering 694000907 bases.

For the comparison, our sparse coding pipeline was then executed under the same settings as in the full cohort analysis (hash size and k-mer size equal to 30 and 31 respectively, and default parameters for the dictionary learning and sparse decomposition of the abundance matrix), but with the number of components matched to the number of bins (225) generated by MetaBat2. To generate Figure 1, the complete genomes retrieved using both approaches were aligned (using nucmer[40] using default parameters) to the individual assemblies of all the samples, and the number of distinct contig hits ($\geq$99% identity and $\geq$ 2500 bp) was recorded.

Comparison of read binning algorithms

The virtual cohort dataset described above and in ref[24] was used to compare the clustering accuracies of the original LSA[13] and sparse coding methods, as well as the performance of directly clustering the columns of the abundance matrix using a k-means algorithm as a baseline.

The reads to genome memberships being comprehensively known in these controlled genome mixtures, clustering accuracy metrics (precision, recall and F-measure) could be quantified as in ref[35] (Table 1). Briefly, each bin is first mapped to its most abundant (in terms of number of reads) genome (note that if each bin is mapped to a single genome, a given genome can be mapped to multiple bins). Precision is defined as the ratio of reads originating from the mapped genome to all the bin's reads. Recall on the other hand reflects how complete a bin is with respect to the sequences of its cognate (mapped) genome. Average precision is the fraction of correctly assigned reads for all assignments to a given cluster averaged over all clusters, while average completeness is averaged over all genomes (including those possibly not assigned to any cluster). We follow ref[35] in order to give larger bins higher weight in performance determinations. Specifically, if X is the set of clusters and Y the set of underlying genomes, precision and recall are defined respectively as:

$$p = \frac{\sum_{x \in X} TP_x}{\sum_{x \in X} TP_x + FP_x} = \frac{\sum_{x \in X} \max_y |x \cap y|}{\sum_{x \in X} |x|}$$

and

$$r = \frac{\sum_{y \in Y} \max_x |x \cap y|}{\sum_{y \in Y} |y|}.$$

The same k-mer abundance matrices (built using a k-mer size of 31 and a number of hash bits (hyperplanes) equal to 30) were used as input for all the methods.

Initial estimate of genome richness and number of components

For the test experiments based on synthetic microbiomes of controlled complexity (e.g. the virtual cohort of 50 individuals, where each microbiome consisted in 100 genomes drawn from a pool of 750 genomes under a given abundance distribution), the number of clusters was set to match the (known) number of distinct genomes segregating in the complete set of samples.

For the analysis of real-world data (e.g. the LifeLinesDeep cohort), where the total number of genotypes is unknown, a meaningful number of components for the sparse decomposition was estimated on the basis of the number of distinct rpS3 ribosomal protein sequences in the analyzed metagenomes (clustered at 98% identity), which roughly corresponds to species level delineations according to ref[41].

Evaluation of read enrichment levels

To assess whether we could identify genomes segregating at consistently low abundance levels in real-life datasets, we characterized the abundance of a dozen of MAGs reconstructed from the LifeLinesDeep cohort analysis by directly mapping the raw reads of the original samples against them. Given the large size of the cohort (and the significant amount of computer resources associated with this analysis), and given that our objective here is to establish whether consistently rare genomes can be identified by the method or not, this analysis was performed on a limited number of genomes.

Relative enrichment levels were estimated by mapping the original reads (after removal of duplicated reads) to the genome-resolved partitions using bwa-mem[39] with default parameters. Uniquely and consistently (i.e. paired) mapped reads were scored to compute enrichment ratios as the number of mapped reads divided by the number of raw reads analyzed, as displayed for example on the x-axes of Figure 4 and the right panels of Figure 5.

Comparison of genome-resolved partitions to reference genomes

To assess the novelty of the genomes assembled from individual partitions produced by our pipeline through the analysis of the LifeLines-Deep cohort, we screened them against two reference libraries. First, the genomes were compared to the Kraken2 (v1) database (https://ccb.jhu.edu/software/kraken2/) built from NCBI's refseq bacteria, archaea, and viral libraries (in August 2018), using the Kraken2 classifier[28] and a confidence score threshold of 0.2. Second, the same genomes were compared against the Human Gastrointestinal Bacteria Genome Collection[6] (HGG, encompassing more than 100 GB of sequence data) using the nucmer aligner[40] with default pa-

rameters. A genome was marked as already known if it shared at least ten distinct 99% identity alignments of length ≥5 kb to any HGG entry.

Binning implementation

Code for the pipeline used to perform the analysis of the LifeLinesDeep cohort can be cloned from https://gitlab.com/kyrgyzov/lsa_slurm, while a more lightweight implementation of key algorithms (including sparse NMF) can be downloaded from https://github.com/vincentprost/LSA_NMF; they draw on the code base of the LSA tool (ref[13] and https://github.com/brian-cleary/LatentStrainAnalysis), and on the SPAMS (SPArse Modeling Software) library that can be downloaded from http://spams-devel.gforge.inria.fr/. The analysis of the metagenomes from the LifeLines-DEEP cohort was carried out on a Bullion S6130 octo module server equipped with 2 Intel Xeon Haswell E7-4890 v3 CPU (18 cores) per module, 8 TB of RAM and 35 TB storage. Most of the tasks being embarassingly parallelizable, they were run through a Slurm workload manager. The analysis took about three weeks wall time, with the sparse decomposition of the k-mer abundance matrix taking less than one day. The bulk of the execution time was spent in pre and post-processing tasks: pre-processing of the 10 terabytes of raw reads to improve load balancing (~5 days), k-mer hashing and counting for constructing the k-mer abundance matrix (~4.5 days), assignments of reads to eigengenomes following the sparse decomposition step (~ 6 days), and assembly of individual read partitions using the spades assembly engine[26] (~2.5 days).

A desirable feature when designing computational pipelines is to have resource requirements, especially memory, scale in a way independent of the sheer data volume. This is the case for the analytical method presented here, as it can be executed "in memory" with the dimensionality of the empirical abundance matrix tailored via the LSH scheme to capture the desired amount of sequence diversity while remaining consistent with the available resource budget. The use of efficient online matrix factorization techniques[15] leads to limited memory footprints. Even though we leveraged here a powerful computer infrastructure to carry out the analysis of the large cohort dataset (10 terabytes of data), our pipeline is routinely executed on commodity hardware for smaller projects.

Availability of source code

Code for the pipeline used in the analysis of the LifeLinesDeep cohort can be cloned from https://gitlab.com/kyrgyzov/lsa_slurm, while a more lightweight implementation of key algorithms (including sparse NMF) can be downloaded from https://github.com/vincentprost/LSA_NMF

Availability of supporting data and materials

Assembled sequences of the genome-resolved bins (more than 50% complete and with less than 5% contamination) recovered from the analysis of the LifeLines-DEEP cohort are available at http://www.genoscope.cns.fr/SCdata/MAGs/. The dataset corresponding to the virtual cohort used in the test experiments can be downloaded from http://www.genoscope.cns.fr/SCdata/vc50/, as well as the reduced spiked dataset used in the preliminary experiment.

Declarations

Consent for publication

Not applicable

Competing Interests

The authors declare no competing financial interests.

Funding

This research was funded by the French Investments for the Future ("Investissements d'Avenir") program FSN-CISN2 (ADAMme project).

Author's Contributions

T.B. conceived the project. O.K., V.P. and T.B. performed the analyses. B.F., S.G. and T.B. supervised the project. T.B. wrote the manuscript, with contributions from O.K. and V.P. All authors approved the final version of the manuscript.

Acknowledgements

The authors wish to thank Brian Cleary and Eric Alm for pioneering pre-assembly binning, Julien Mairal and Ghislain Durif for developing the spams library and for useful discussions, Alexandre d'Aspremont for helpful insights and two reviewers for much useful feedback.

References

1. Castelle CJ, Banfield JF. Major new microbial groups expand diversity and alter our understanding of the tree of life. Cell 2018;172(6):1181–1197.
2. Parks DH, Rinke C, Chuvochina M, Chaumeil PA, Woodcroft BJ, Evans PN, et al. Recovery of nearly 8,000 metagenome-assembled genomes substantially expands the tree of life. Nature microbiology 2017;2(11):1533.
3. Pasolli E, Asnicar F, Manara S, Zolfo M, Karcher N, Armanini F, et al. Extensive unexplored human microbiome diversity revealed by over 150,000 genomes from metagenomes spanning age, geography, and lifestyle. Cell 2019;.
4. Almeida A, Mitchell AL, Boland M, Forster SC, Gloor GB, Tarkowska A, et al. A new genomic blueprint of the human gut microbiota. Nature 2019;p. 1.
5. Nayfach S, Shi ZJ, Seshadri R, Pollard KS, Kyrpides N. Novel insights from uncultivated genomes of the global human gut microbiome. Nature 2019;p. 1.
6. Forster SC, Kumar N, Anonye BO, Almeida A, Viciani E, Stares MD, et al. A human gut bacterial genome and culture collection for improved metagenomic analyses. Nature biotechnology 2019;37(2):186.
7. Kallus Y, Miller JH, Libby E. Paradoxes in leaky microbial trade. Nature communications 2017;8(1):1361.
8. Jousset A, Bienhold C, Chatzinotas A, Gallien L, Gobet A, Kurm V, et al. Where less may be more: how the rare biosphere pulls ecosystems strings. The ISME journal 2017;11(4):853.
9. Kalenitchenko D, Le Bris N, Peru E, Galand PE. Ultrarare marine microbes contribute to key sulphur-related ecosystem functions. Molecular ecology 2018;27(6):1494–1504.

10. Benjamino J, Lincoln S, Srivastava R, Graf J. Low-abundant bacteria drive compositional changes in the gut microbiota after dietary alteration. Microbiome 2018;6(1):86.
11. Wu YW, Ye Y. A novel abundance-based algorithm for binning metagenomic sequences using l-tuples. Journal of Computational Biology 2011;18(3):523–534.
12. Yang B, Peng Y, Leung HCM, Yiu SM, Chen JC, Chin FYL. Unsupervised binning of environmental genomic fragments based on an error robust selection of l-mers. In: BMC bioinformatics, vol. 11 BioMed Central; 2010. p. S5.
13. Cleary B, Brito IL, Huang K, Gevers D, Shea T, Young S, et al. Detection of low-abundance bacterial strains in metagenomic datasets by eigengenome partitioning. Nature biotechnology 2015;33(10):1053.
14. Řehůřek R, Sojka P. Software Framework for Topic Modelling with Large Corpora. In: Proceedings of the LREC 2010 Workshop on New Challenges for NLP Frameworks Valletta, Malta: ELRA; 2010. p. 45–50. http://is.muni.cz/publication/884893/en.
15. Mairal J, Bach F, Ponce J, Sapiro G. Online learning for matrix factorization and sparse coding. Journal of Machine Learning Research 2010;11(Jan):19–60.
16. Kang DD, Froula J, Egan R, Wang Z. MetaBAT, an efficient tool for accurately reconstructing single genomes from complex microbial communities. PeerJ 2015;3:e1165.
17. Kang D, Li F, Kirton ES, Thomas A, Egan RS, An H, et al. MetaBAT 2: an adaptive binning algorithm for robust and efficient genome reconstruction from metagenome assemblies. PeerJ Preprints 2019;7:e27522v1.
18. Zhernakova A, Kurilshikov A, Bonder MJ, Tigchelaar EF, Schirmer M, Vatanen T, et al. Population-based metagenomics analysis reveals markers for gut microbiome composition and diversity. Science 2016;352(6285):565–569.
19. Alneberg J, Bjarnason BS, De Bruijn I, Schirmer M, Quick J, Ijaz UZ, et al. Binning metagenomic contigs by coverage and composition. Nature methods 2014;11(11):1144.
20. Chatterji S, Yamazaki I, Bai Z, Eisen JA. CompostBin: A DNA composition-based algorithm for binning environmental shotgun reads. In: Annual International Conference on Research in Computational Molecular Biology Springer; 2008. p. 17–28.
21. Van Lang T, Van Hoai T, et al. A two-phase binning algorithm using l-mer frequency on groups of non-overlapping reads. Algorithms for Molecular Biology 2015;10(1):2.
22. Girotto S, Pizzi C, Comin M. MetaProb: accurate metagenomic reads binning based on probabilistic sequence signatures. Bioinformatics 2016;32(17):i567–i575.
23. Tringe SG, Von Mering C, Kobayashi A, Salamov AA, Chen K, Chang HW, et al. Comparative metagenomics of microbial communities. Science 2005;308(5721):554–557.
24. Gkanogiannis A, Gazut S, Salanoubat M, Kanj S, Brüls T. A scalable assembly-free variable selection algorithm for biomarker discovery from metagenomes. BMC bioinformatics 2016;17(1):311.
25. Charikar MS. Similarity estimation techniques from rounding algorithms. In: Proceedings of the thiry-fourth annual ACM symposium on Theory of computing ACM; 2002. p. 380–388.
26. Bankevich A, Nurk S, Antipov D, Gurevich AA, Dvorkin M, Kulikov AS, et al. SPAdes: a new genome assembly algorithm and its applications to single-cell sequencing. Journal of computational biology 2012;19(5):455–477.
27. Parks DH, Imelfort M, Skennerton CT, Hugenholtz P, Tyson GW. CheckM: assessing the quality of microbial genomes recovered from isolates, single cells, and metagenomes. Genome research 2015;25(7):1043–1055.
28. Wood DE, Salzberg SL. Kraken: ultrafast metagenomic sequence classification using exact alignments. Genome biology 2014;15(3):R46.
29. Luo C, Knight R, Siljander H, Knip M, Xavier RJ, Gevers D. ConStrains identifies microbial strains in metagenomic datasets. Nature biotechnology 2015;33(10):1045.
30. Pell J, Hintze A, Canino-Koning R, Howe A, Tiedje JM, Brown CT. Scaling metagenome sequence assembly with probabilistic de Bruijn graphs. Proceedings of the National Academy of Sciences 2012;109(33):13272–13277.
31. Brown CT, Moritz D, O'brien M, Reidl F, Reiter T, Sullivan B. Exploring neighborhoods in large metagenome assembly graphs reveals hidden sequence diversity. BioRxiv 2019;p. 462788.
32. Albertsen M, Hugenholtz P, Skarshewski A, Nielsen KL, Tyson GW, Nielsen PH. Genome sequences of rare, uncultured bacteria obtained by differential coverage binning of multiple metagenomes. Nature biotechnology 2013;31(6):533.
33. Wu YW, Tang YH, Tringe SG, Simmons BA, Singer SW. MaxBin: an automated binning method to recover individual genomes from metagenomes using an expectation-maximization algorithm. Microbiome 2014;2(1):26.
34. Lu YY, Chen T, Fuhrman JA, Sun F. COCACOLA: binning metagenomic contigs using sequence COmposition, read CoverAge, CO-alignment and paired-end read LinkAge. Bioinformatics 2017;33(6):791–798.
35. Meyer F, Hofmann P, Belmann P, Garrido-Oter R, Fritz A, Sczyrba A, et al. AMBER: assessment of metagenome binners. GigaScience 2018;7(6):giy069.
36. Koslicki D, Falush D. MetaPalette: A K-mer painting approach for metagenomic taxonomic profiling and quantification of novel strain variation. MSystems 2016;1(3):e00020-16.
37. Efron B, Hastie T, Johnstone I, Tibshirani R, et al. Least angle regression. The Annals of statistics 2004;32(2):407–499.
38. Sharon I, Morowitz MJ, Thomas BC, Costello EK, Relman DA, Banfield JF. Time series community genomics analysis reveals rapid shifts in bacterial species, strains, and phage during infant gut colonization. Genome research 2013;23(1):111–120.
39. Li H, Durbin R. Fast and accurate long-read alignment with Burrows-Wheeler transform. Bioinformatics 2010;26(5):589–595.
40. Marçais G, Delcher AL, Phillippy AM, Coston R, Salzberg SL, Zimin A. MUMmer4: a fast and versatile genome alignment system. PLoS computational biology 2018;14(1):e1005944.
41. Sharon I, Morowitz MJ, Thomas BC, Costello EK, Relman DA, Banfield JF. Time series community genomics analysis reveals rapid shifts in bacterial species, strains, and phage during infant gut colonization. Genome research 2013;23(1):111–120.

Table 4. Supplementary Table

cluster id	# of spiked reads	# of reads	ratio
0	2782	5106	0.544849
1	0	6164	0.000000
2	0	7284	0.000000
3	0	4352	0.000000
4	0	5774	0.000000
5	6	6594	0.000910
6	0	4498	0.000000
7	0	5134	0.000000
8	0	6380	0.000000
9	0	7206	0.000000
10	0	5490	0.000000
11	0	6220	0.000000
12	0	6574	0.000000
13	0	6252	0.000000
14	0	6230	0.000000
15	0	6624	0.000000
16	0	5600	0.000000
17	8	6704	0.001193
18	2	6802	0.000294
19	2	67812	0.000029

Cluster assignments of reads from a target genome versus background (unrelated) reads. Nearly all the 2800 reads from the target genome segregating at low levels in the samples (100 paired-reads per sample in 14 samples, none in the remaining samples) are binned in a single partition using our bin-first pipeline, leading to the complete genome after assembly. No kilobase-sized contig could be assembled from any individual sample, making the assembly-first protocol inoperable (see main text).

Figure 1

Click here to download Figure SuppTableNbar3d.pdf

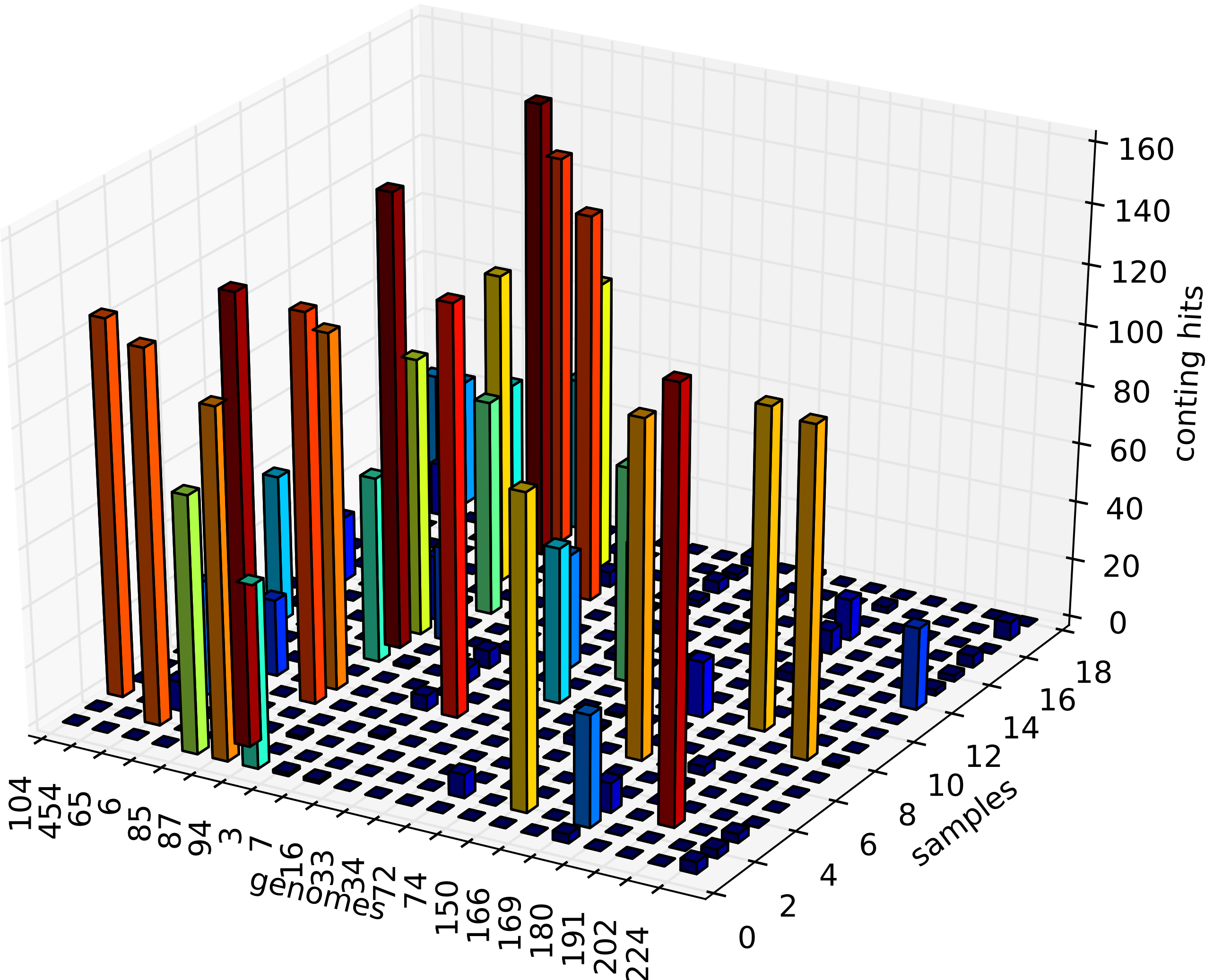

Figure 2A

Click here to download Figure strains1.png

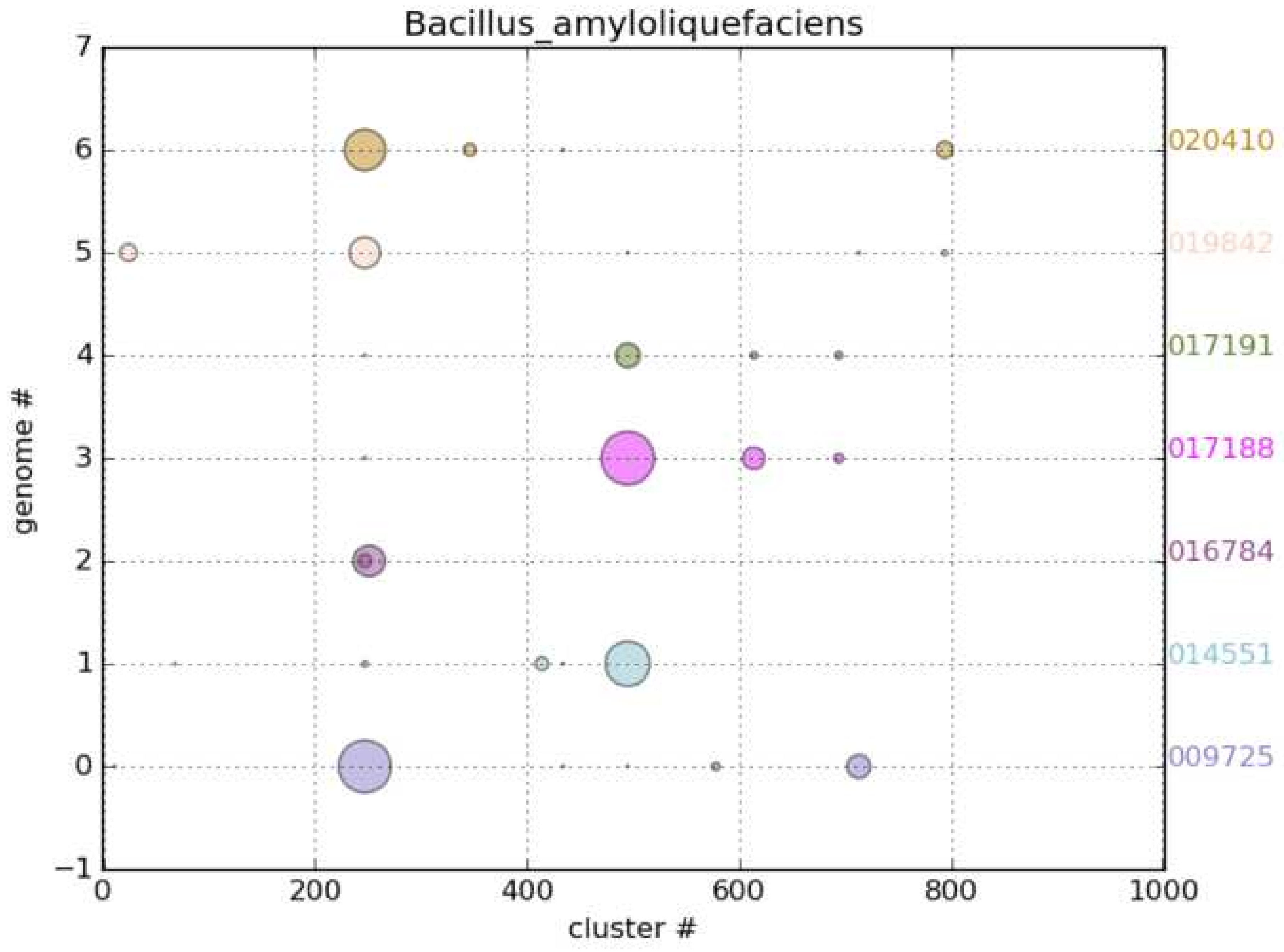

Figure 2B

Click here to download Figure strains3.png

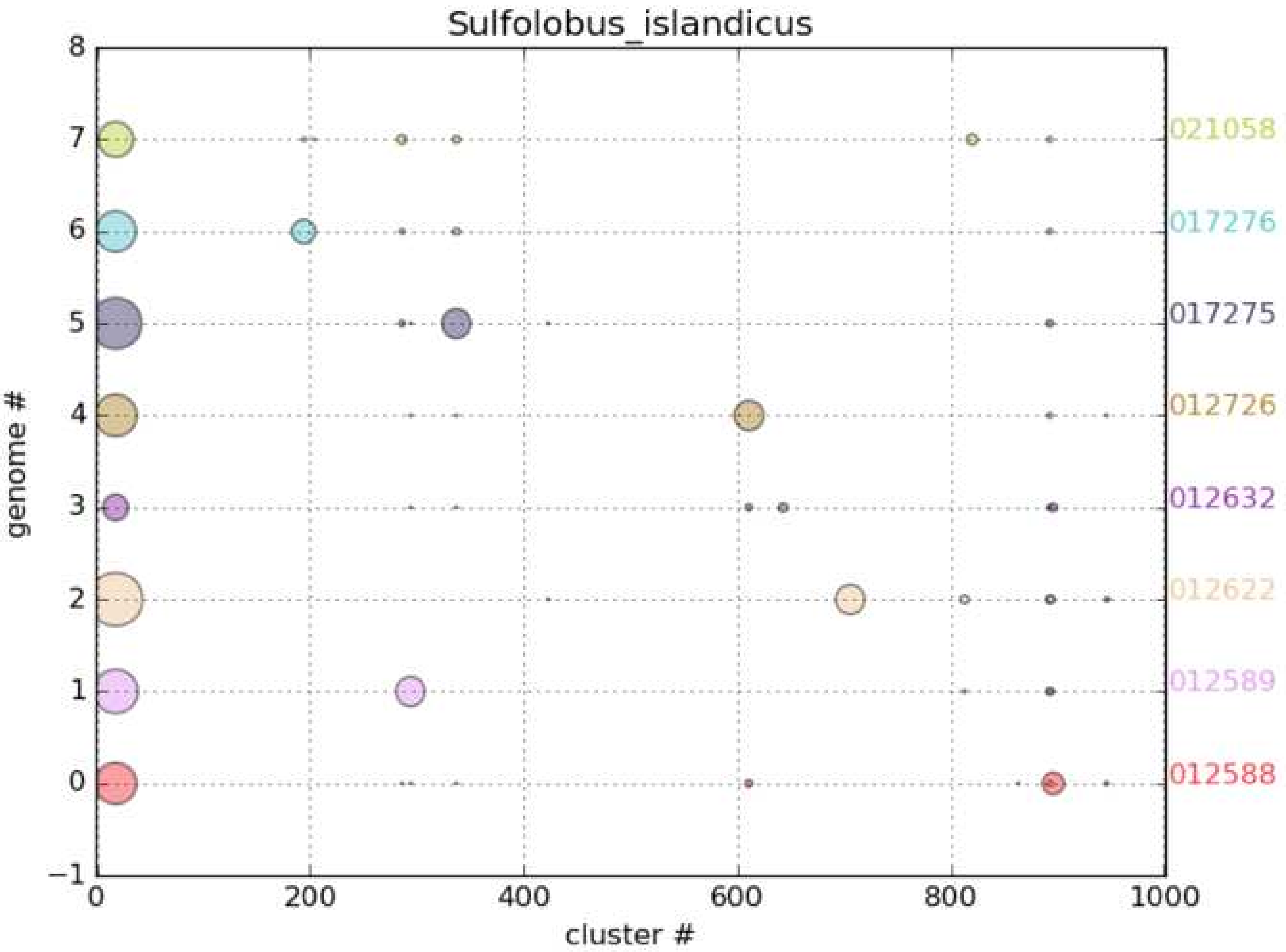

Figure 3

Click here to download Figure psz_histo.png

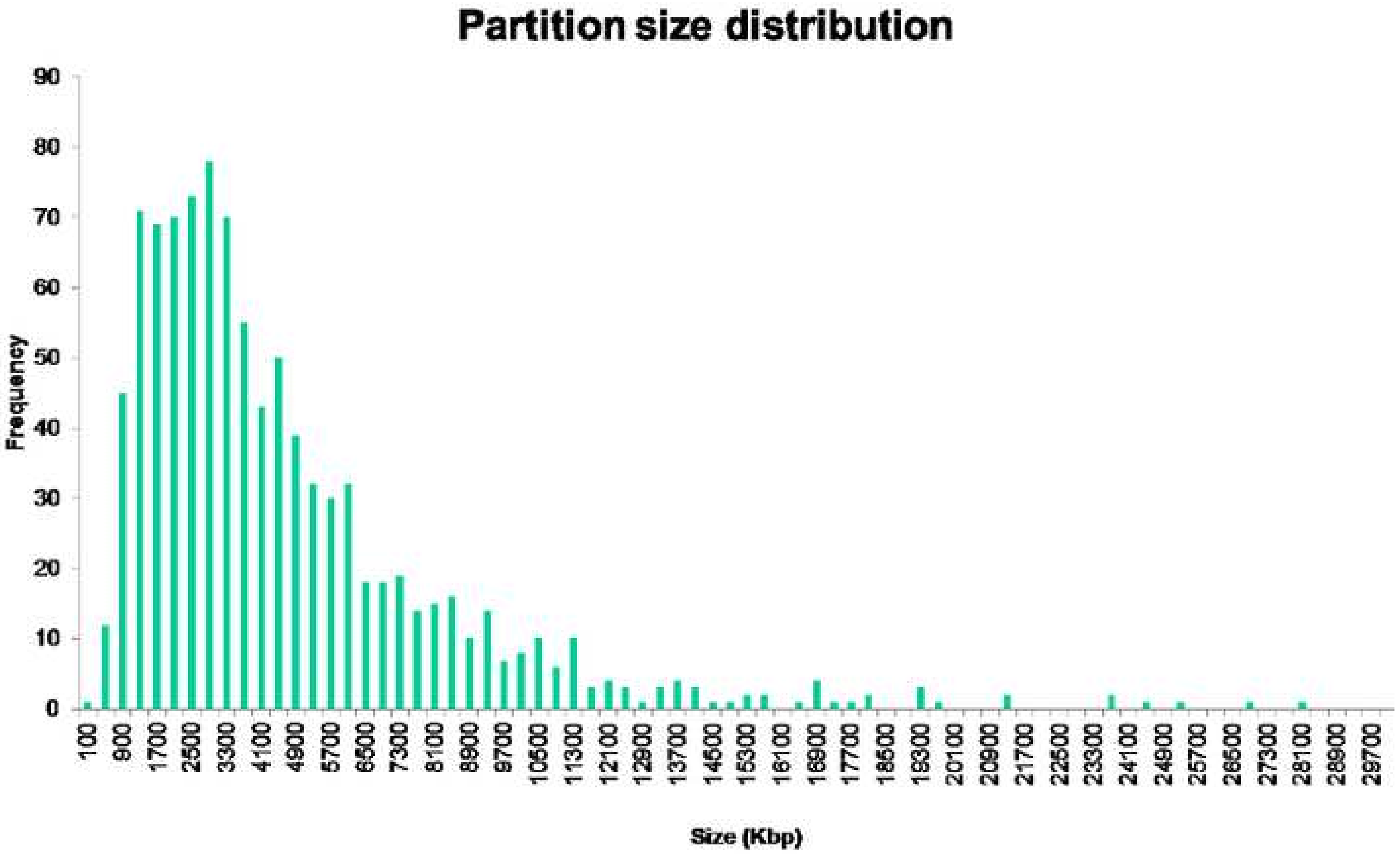

Figure 4A

Click here to download Figure enrich_rare.png

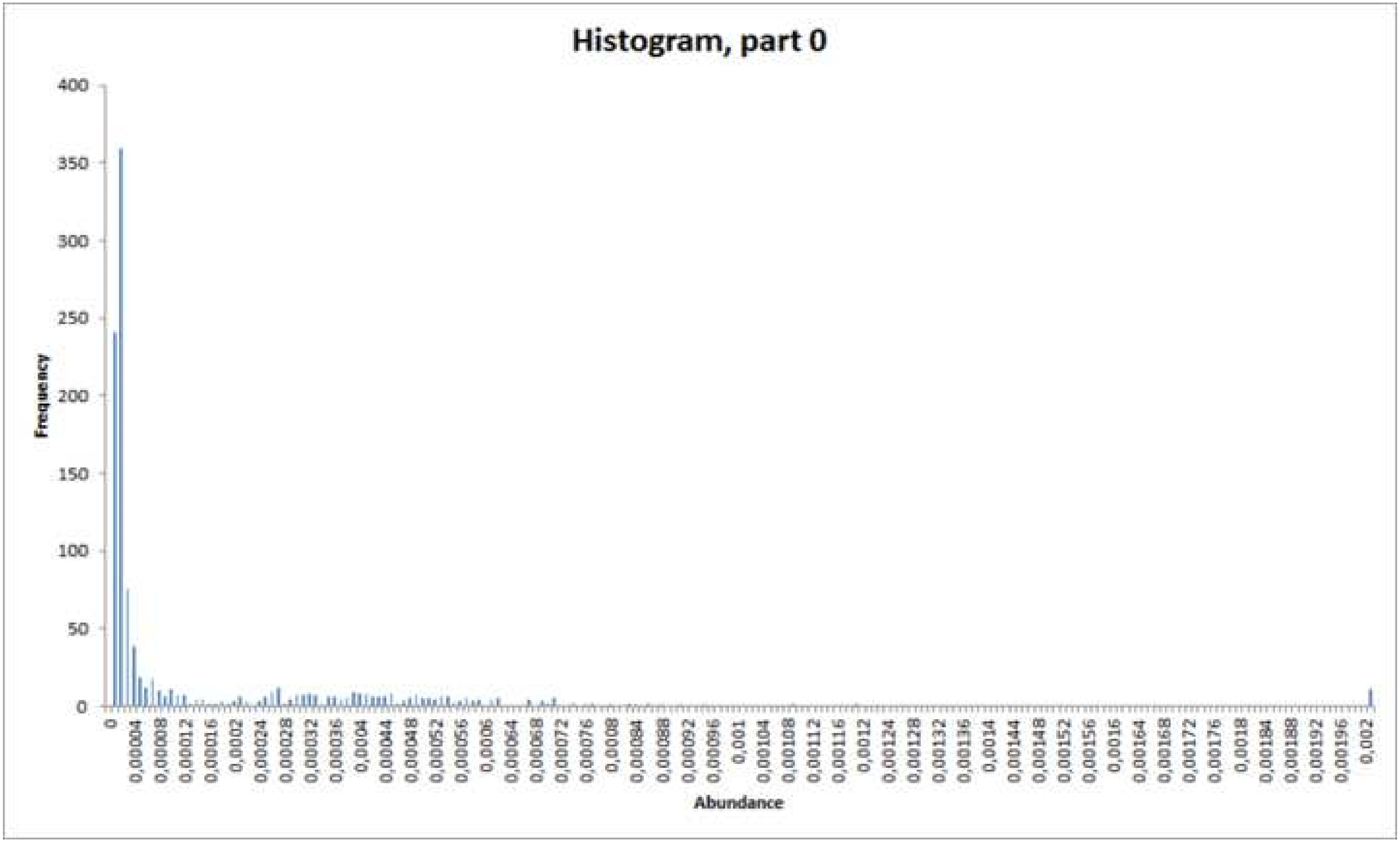

Figure 4B

Click here to download Figure enrich_abund.png

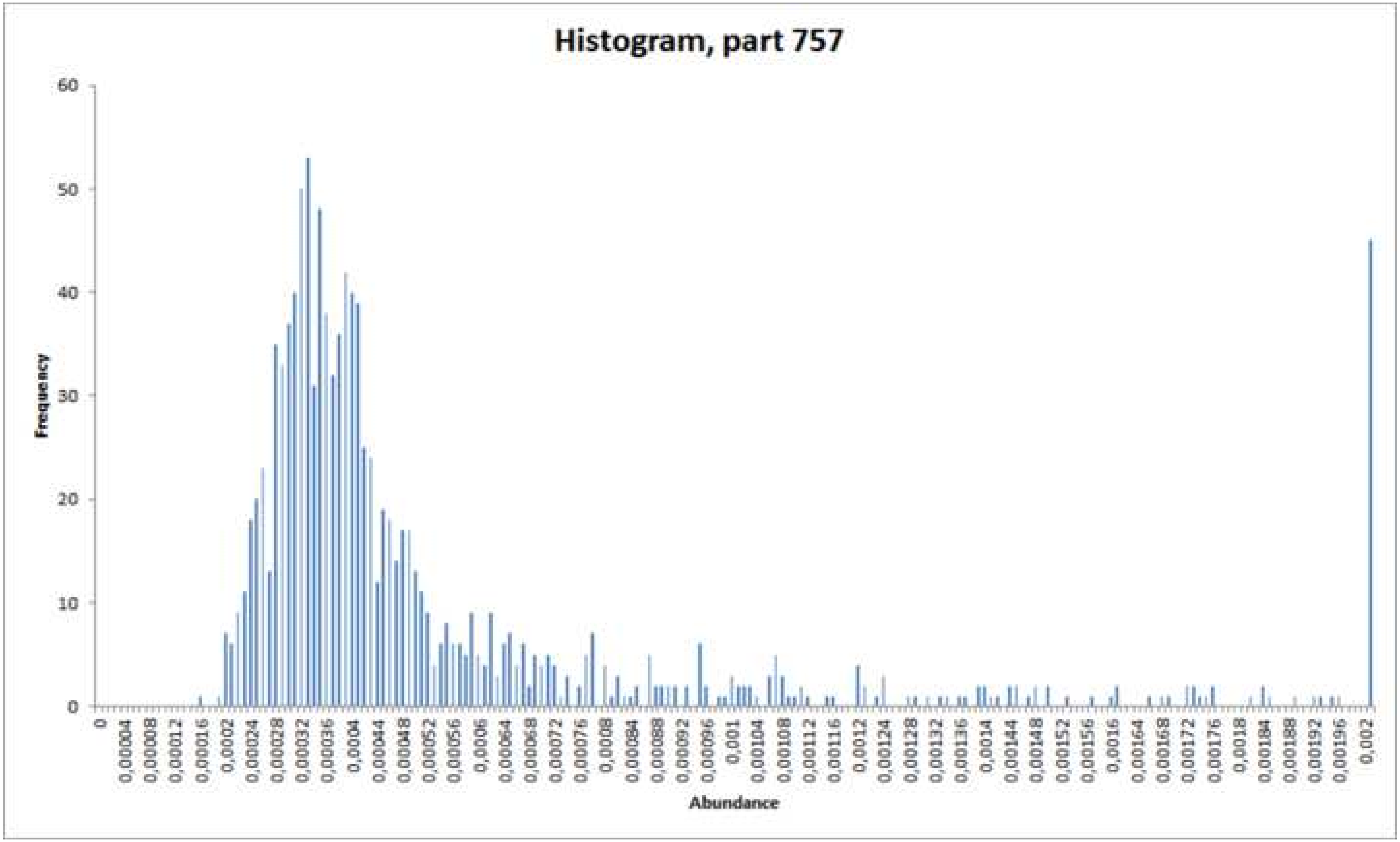

Figure 5 A

Click here to download Figure bact_470_2_gc_cov.pdf

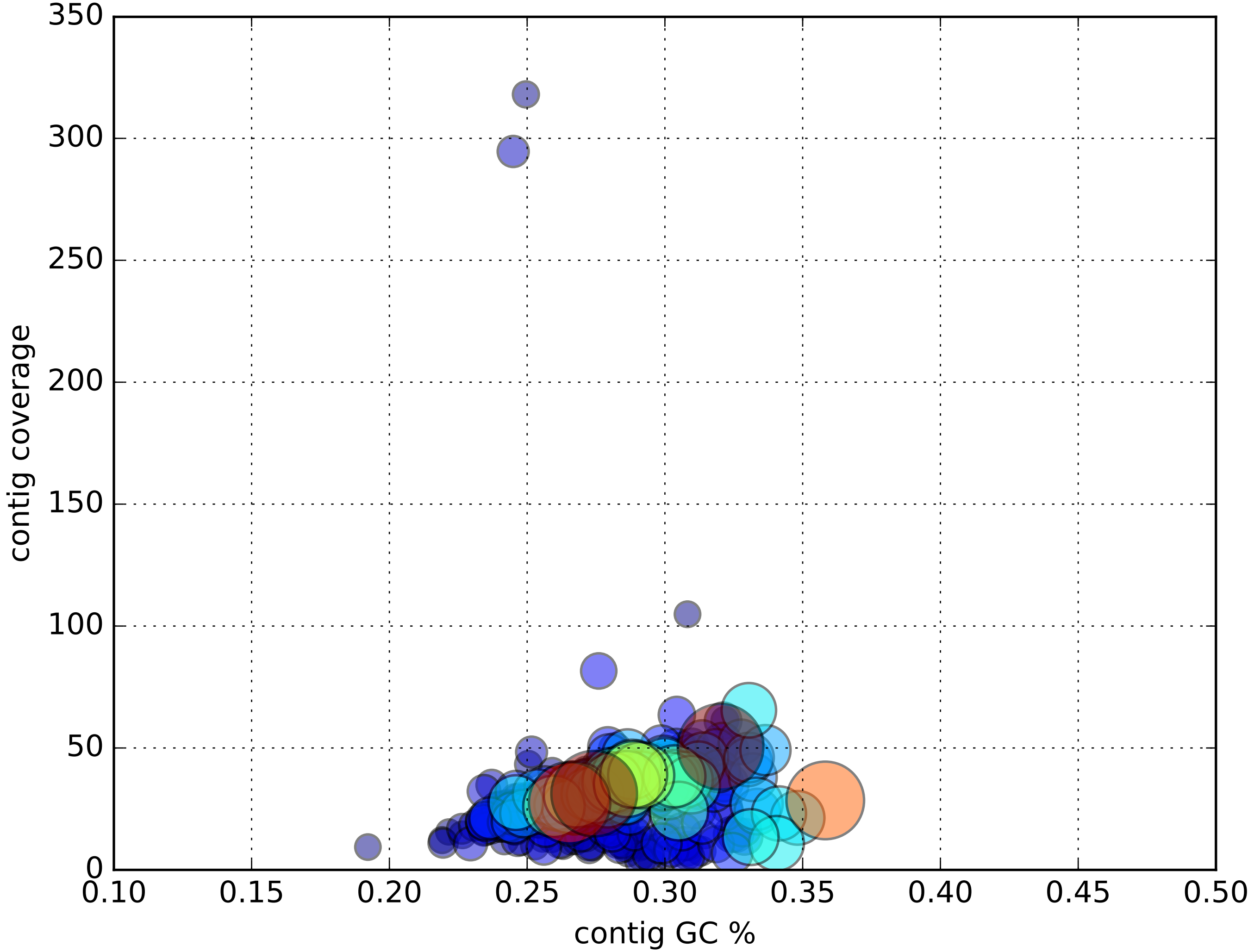

Figure 5 B

Click here to download Figure bact_470_2_histo.png

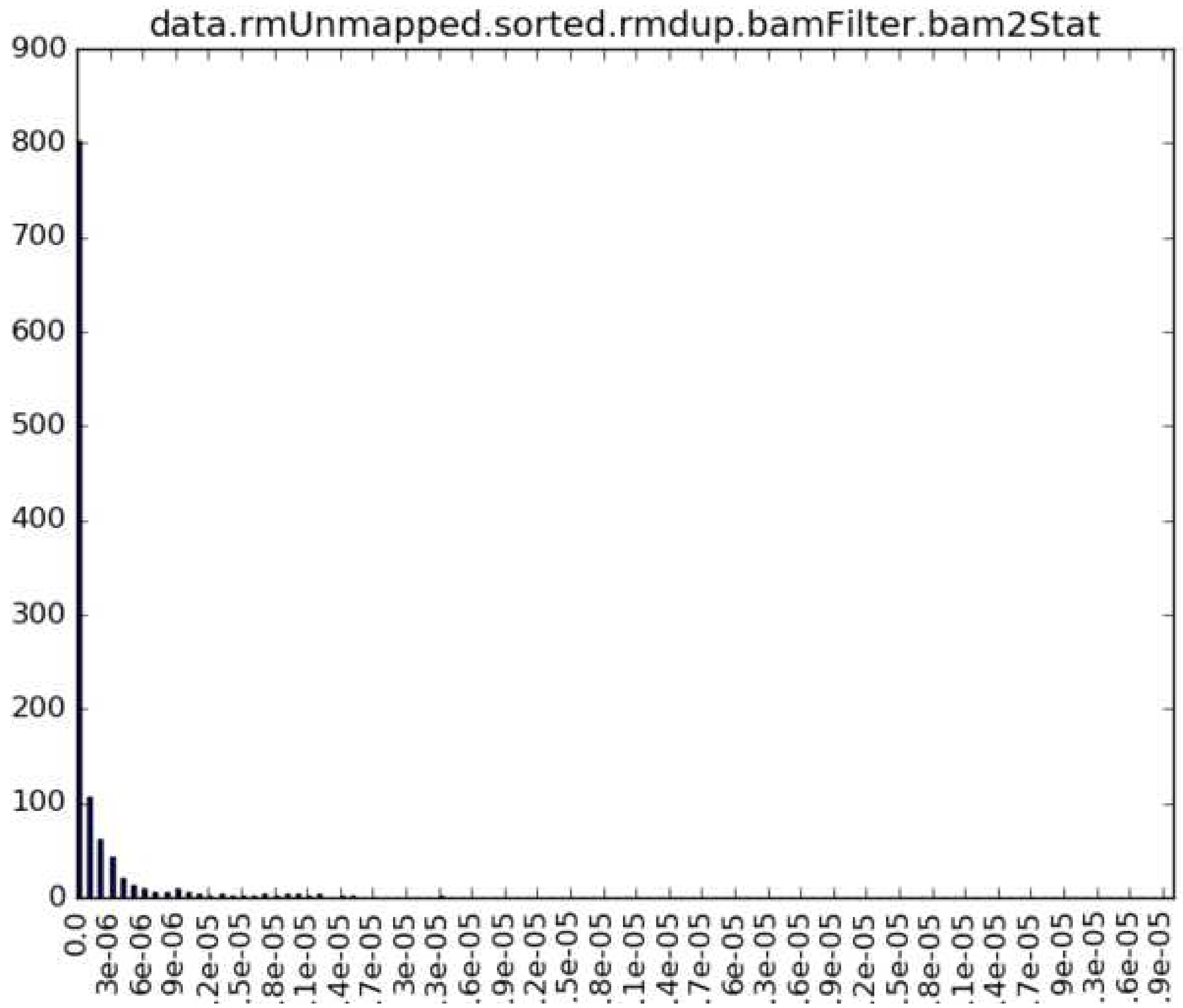

Figure 5 C

Click here to download Figure bact_766_0_gc_cov.pdf

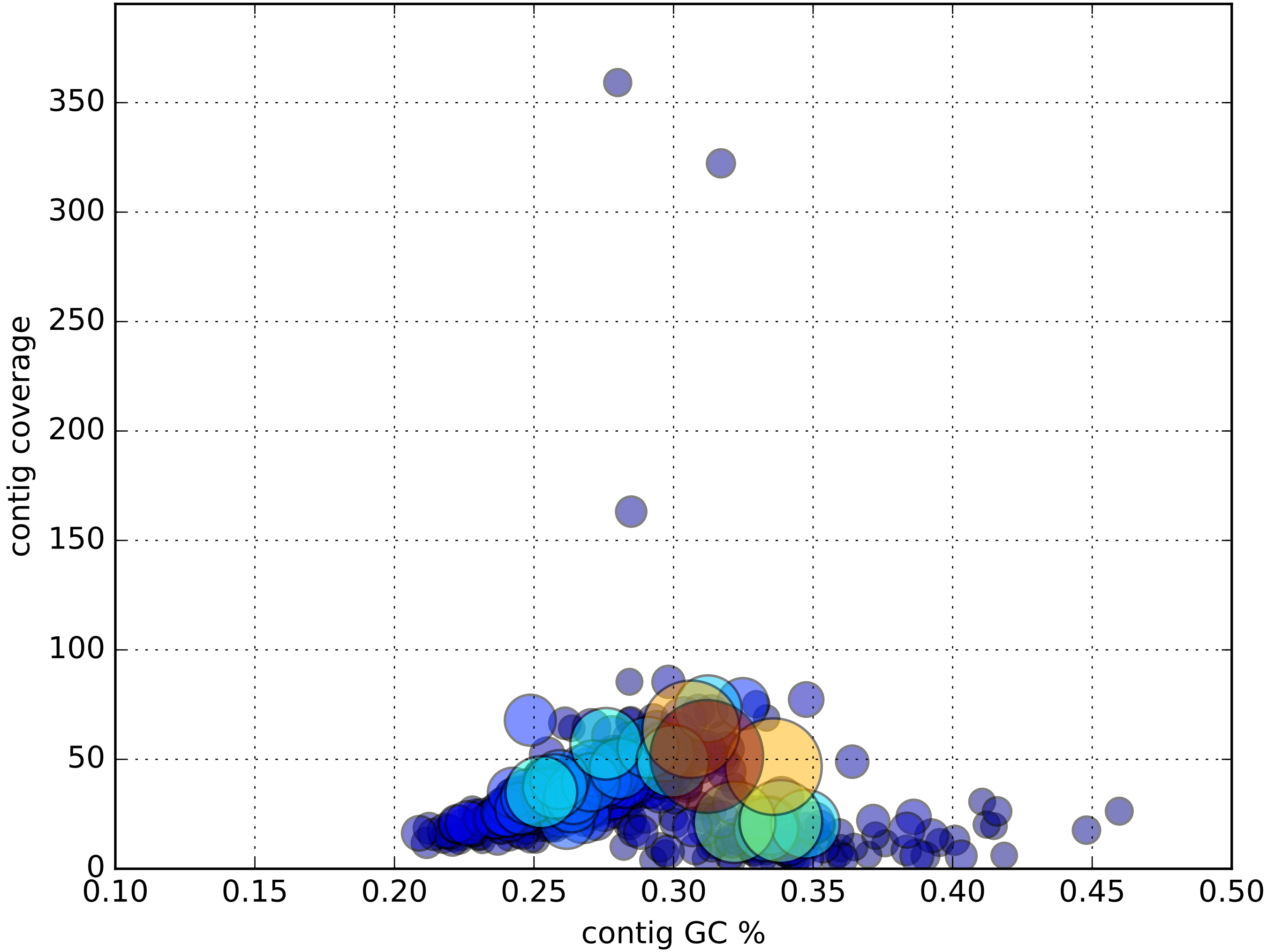

Figure 5 D

Click here to download Figure bact_766_0_histo.png

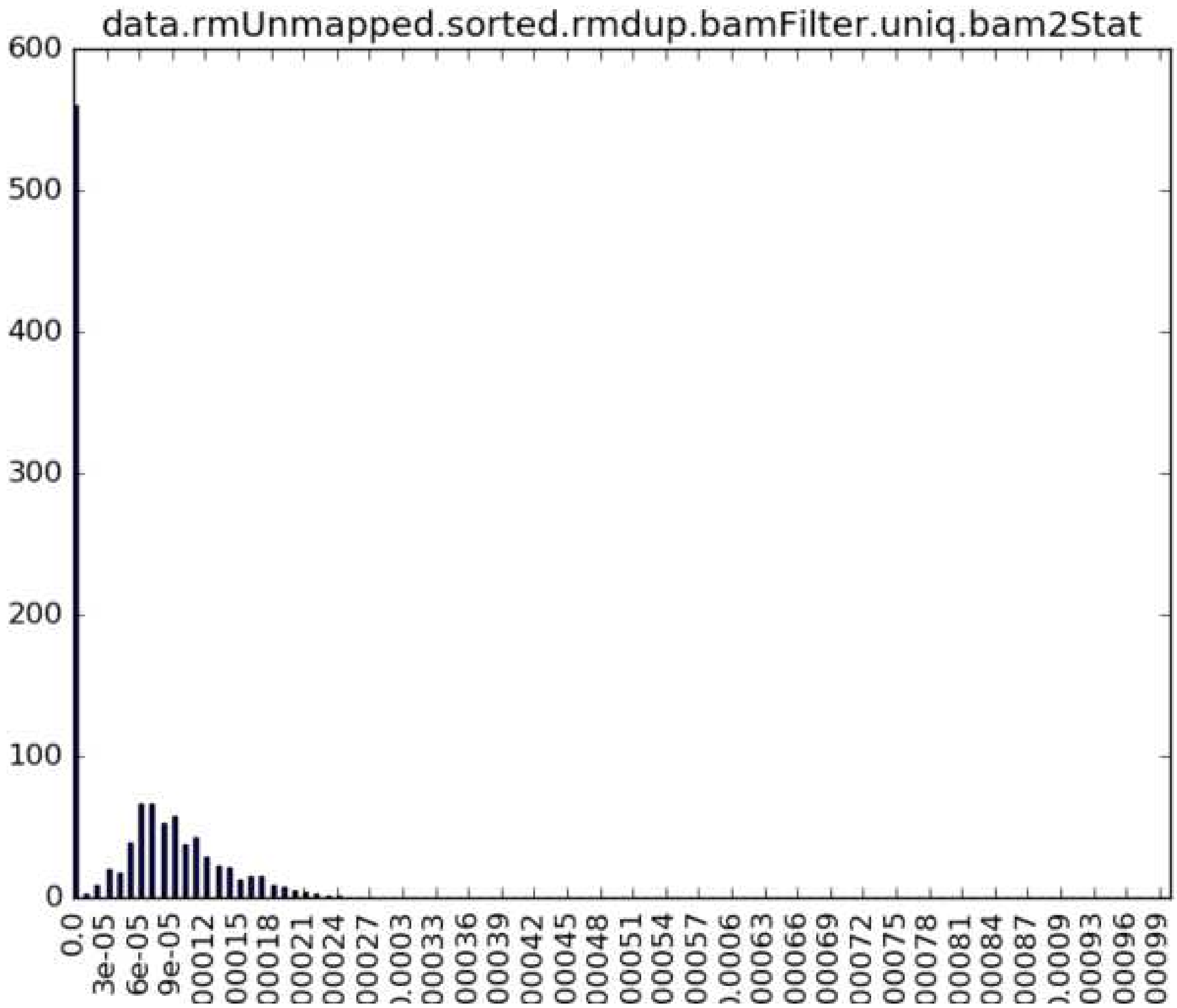

Manuscript
Click here to download Personal Cover Response_to_Reviewers.pdf

Response to reviewers:

This recrafted manuscript includes and discusses results from two additional experiments suggested by the reviewers. Because of their importance in the revised manuscript, we present them briefly before specifically addressing the comments of the reviewers afterwards.

The first additional experiment involved a synthetic minimal setup aiming to compare the ability of bin-first versus assembly-first strategies to aggregate rare reads across samples, while the second is a real-life experiment aiming to measure the efficiency of both approaches in terms of complete genomes recovery, starting with a collection of real human gut metagenomes.

We briefly describe these two experiments hereunder.

i) The first additional experiment illustrates a situation where assembly-first approaches are not able to recover a target genome (because target genome sequences are too low in number in any single sample, see below) whereas a bin-first approach is successful at it.

The experimental setup involved distributing a very low number of short reads (100 paired reads) randomly sampled from a target genome (a 10 kbp plasmid) into 14 samples containing a background of 20000 unrelated bacterial sequences each (4 further samples contained only background sequences with no read from the target genome at all). No single kilobase-sized fragment could be recovered by assembling the sequences from each sample individually, hence precluding the application of assembly-first methods (e.g. contig binning methods like metabat require >= 1500 bp sequences as input). On the other hand, more than 99% of the reads originating from the target genome could be segregated in a single cluster/bin based on abundance covariance (Supplementary Table 1), leading to the complete recovery of the target genome in a single contig after assembly.

ii) The second experiment aimed at directly comparing the genome recovery yield of assembly-first versus bin-first strategies on a real-life dataset. We selected the raw sequence data from 18 (randomly chosen) individuals of the LifeLinesDeep cohort, and either assembled these individually (i.e. on a sample by sample basis, with metaSPAdes) followed by contig binning across samples with the MetaBat2 adaptive algorithm, or clustered the raw reads using our read-level binning pipeline, followed by metaSPAdes assembly of the resulting partitions/bins.

Fourteen nearly (> 90%) complete and uncontaminated (< 5%) genomes were recovered using the assembly-first approach, versus 7 using the bin-first method.

Crucially, the two genome sets are disjoint, with no complete genome recovered by both approaches. The fact that the bin-first approach was able to recover a significant number of complete genomes not identified by the assembly-first approach (and reciprocally) illustrates a form of complementarity between the two strategies.

We should briefly note that the number of samples (18) used in this experiment is relatively low, and that related approaches based on abundance-covariance, like Concoct or LSA among others, require a higher number of samples to achieve best performance (about 50 samples for concoct and from 30 to 50 for LSA).

Reviewer #1: This manuscript describes a reads clustering algorithm that utilized sparse coding on k-mer covariance. If focusing on the reads clustering algorithm alone, this manuscript may be an interesting read; there are however other fallacies that can still be improved. Here are my reviews.

1. In general the authors are trying to establish the statement that contig clustering is not good enough; therefore we need to perform clustering at the reads level. The rationale behind this statement is that low abundance genome cannot be assembled and hence cannot be binned very well. On one hand this statement seems to hold and was mentioned in a few papers. This reason alone, however, does not justify the excessive computational resources that are required in the reads clustering process. I suggest the authors to at least compare reads and contig clustering results to show that, yes, their reads clustering algorithm indeed unearth more genomes (especially less abundant ones) than contig-based methods.

We see three distinct items in this dense remark that we think might be worth answering separately and sequentially hereunder:

1.1)

"In general the authors are trying to establish the statement that contig clustering is not good enough; therefore we need to perform clustering at the reads level. The rationale behind this statement is that low abundance genome cannot be assembled and hence cannot be binned very well. On one hand this statement seems to hold and was mentioned in a few papers."

First, we need to clearly state that we do not consider read-level clustering as a panacea able to both address limitations of contig binning while at the same time surpassing it in the application domain where it excels (i.e. recovering genomes of high (i.e. about 10x) abundance).

Instead, we try in the present work to make the case for the complementary nature of both approaches, by showing the ability of the bin-first approach i) to recover low-abundance genomes not accessible to assembly-first approaches (first additional experiment, and section on enrichment measurements and Figure 4), ii) to recover relatively abundant (i.e. yielding kilobase-sized contigs in individual sample assemblies) genomes not identified by assembly-first approaches (additional experiment 2).

We believe that the application domain of read-level binning is much narrower than contig clustering, with the latter much efficiently allowing picking low-hanging fruits. In our opinion the main appeal of read-binning resides in its ability to access consistently lower-abundance genomes. In that sense, and as pointed by the second referee, the method is actually more close to the motivation of single-cell techniques.

On the other hand, as the algorithmic core of bin-first versus assembly-first strategies differs so much, we did not expect the results of any of them to be a superset of the other. Instead, we demonstrate in the new version of the manuscript that the genome sets recovered by both approaches show limited overlap, and therefore that both methods should be viewed as complementary rather than antagonistic.

This belief is actually the main reason why we did not initially compare our bin-first approach to assembly-first ones, but instead focused on comparing its read clustering accuracy to the state of the art read-binning algorithm LSA. In this respect, it is worth noting that the publication of the latter (Nature Biotechnol. 2015, 33(10):1053-60) did not involve any comparison against assembly-first methods.

1.2)

"This reason alone, however, does not justify the excessive computational resources that are required in the reads clustering process."

Regarding the computational cost of the read clustering process, we would like to stress that there are no intrinsic impediment to run the read-binning pipeline on commodity hardware, and that the use of the Sequana Bull machine was exclusively motivated by the sheer size (10 terabytes) of the dataset analyzed.

On one hand, the key algorithmic step of decomposing potentially large k-mer abundance matrices can be streamed and/or carried out iteratively using so-called online techniques, thus precluding the need to map the full k-mer abundance matrix into memory.

It could also be worth mentioning that it is the availability of efficient streaming libraries for the factorization of the k-mer abundance matrix that drove the authors of the LSA method towards using a rank-reduced singular value decomposition (B. Cleary and E. Alm, personal communication).

Instead, we were keen to cast the read binning problem in a sparse coding framework in order to be able to enforce (biologically sound) sparsity and non-negativity constraints inherent to k-mer count data, and decided to make use of the SPAMS library to carry out online sparse matrix factorization.

On the other hand, we acknowledge that the computational requirements for bin-first strategies are higher than for assembly-first ones (unless maybe the latter require a large cross-assembly computation.

However, several techniques can be leveraged to handle the increased computational demand. Beyond iterative and online matrix factorization techniques mentioned above, locality sensitive hashing (LSH) is a key ingredient that allows us to control the dimensionality of the k-mer space (reducing it from 4^k to 2^h, where k is the k-mer size and h is the number of hash bits). The efficiency of LSH for k-mer indexing and counting was previously demonstrated on a terabase-sized dataset (Cleary et al., Nat. Biotechnol. 2015), which prompted us to exploit it for the processing of an order of magnitude larger dataset (the microbiomes from the LifeLinesDeep cohort).

Thus, even though the possibility offered by Bull's Sequana platform to benefit from in-memory computing for the *joint* analysis of the large cohort was an important initial motivation, we should make clear that the pipeline itself is not bound to mass-memory architectures, even for very large (terabyte-sized) k-mer matrices.

The reason we used the Bull hardware here was to demonstrate the scalability of the complete pipeline by processing a 10 terabytes dataset (by scalability, we mean the ability of the method to adapt to order of magnitude change in the input, and its ability to maintain its functionality and performance under high demand).

As a matter of fact, we did perform the new experiments described in the revised manuscript on a commodity hardware (Dell PowerEdge R910).

We should also stress that pipeline steps like sequence hashing, k-mer counting or read partitioning among others, are embarrassingly parallel by nature.

1.3)

"I suggest the authors to at least compare reads and contig clustering results to show that, yes, their reads clustering algorithm indeed unearth more genomes (especially less abundant ones) than contig-based methods."

We did perform an additional experiment in order to directly compare assembly-first versus bin-first approaches in terms of complete genome recovery.

The raw sequence data from 18 (randomly chosen) individuals of the LLDeep cohort were either assembled individually (i.e. on a sample by sample basis) with metaSPAdes (v3.13.0) followed by contig binning across samples with the MetaBat2 adaptive algorithm, or the raw reads were clustered using our read-level binning pipeline, followed by metaSPAdes assembly of the resulting partitions/bins.

This led to fourteen nearly complete genomes being recovered using the assembly-first approach, versus 7 using the bin-first method, with no genome being recovered by both methods.

The genomes recovered by the bin-first approach cannot be described as low-abundance, as significant portions of them were already assembled in individual samples. The surprising lack of overlap between the two genome sets in this experiment is thus not attributable to fundamental differences in abundance levels between the genomes recovered by the two approaches.

We assessed potential differences between the distributions of binned genome sequences across the original samples. This highlighted distinct patterns for the two approaches, with the genomes from the bin-first approach aggregating sequences from a larger number of samples (see new Figure 1). The number of contigs clustered per genome bin, and to a lesser extent the average contig coverage, also differs between the two approaches.

These results are consistent with the notion that –in this experiment- the assembly-first approach targeted genomes for which the abundance covariation signal was weaker, making the task of the bin-first approach more difficult. This is

consistent with the observation that sequences from genomes produced through the assembly-first approach were frequently located in large (dozens of Mbp in size) and unresolved partitions computed by read-level binning.

Among the 14 genomes recovered by the assembly-first approach, three were not represented in the set of 164 MAGs recovered from the entire cohort in our initial analysis. More surprisingly, 4 out of the 7 complete genomes recovered by our bin-first pipeline from the analysis of 18 samples were not represented among the MAGs established from the full cohort analysis, indicative of a lack of stability of the method that we relate in the discussion to bin fragmentation provoked by the occurrence of strain-level variation across the samples/individuals.

Overall, and despite these limitations, this experiment suggests the two approaches can benefit from each other in terms of the genome profiles recovered, hence are better viewed as complementary.

2. From the ecological point-of-view, rare species tend to contribute less to the microbial community (e.g. Bello 2007 Community Biology). This viewpoint of course is still up to certain debate and may not hold everywhere, but it (and the lack of counter statement) also undermines the necessity to extract very low abundance genomes except in the view of cataloguing species. Maybe the authors can discuss a bit about this aspect regarding to why they design something explicitly for low abundance genomes.

On the theoretical side, we could invoke results from the modeling of microbial trade of diffusible goods. For example, the so-called "curse of increased efficiency" in Kallus et al., describes an apparent paradox where one bacterial species becomes rarer in the population even though it becomes fitter and more efficient at producing a key metabolic resource. This is provoked by metabolic interdependencies that can evolve via trade in microbial consortia, and that can lead to low-abundance organisms becoming essential for a faster growth rate of the community.

On the experimental side, we refer to the general discussion of Jousset at al., and to two studies documenting the role of "ultra-rare" bacteria in ecosystem-level productivity and of low abundance bacteria in driving termite's hindgut bacterial community composition.

3. The evaluation was only conducted on their own datasets. I suggest the authors to try their method on other well-established datasets such as CAMI-Challenge.

We did look at the CAMI datasets at the time we started evaluating our method, but these were essentially build for benchmarking taxonomic profiling and binning methods (with the latter almost implicitly referring to assembly-first methods). Even though the overall numbers and types of datasets are substantial, any single dataset only consisted in a relatively small number (up to 12) of samples.

For example, the second CAMI Human Microbiome Project Dataset consists in simulated metagenome data from five different body sites of the human host, namely gastrointestinal tract, oral cavity, airways, skin and urogenital tract. Even though the total number of samples is 49, there are only 10 samples for each site (actually 9 for the urogenital tract), which is not really appropriate for evaluating our read-level binning exploiting abundance-covariation across larger cohorts.

The so-called "Strain Madness Dataset" dataset is the only dataset featuring a large number (100) of samples, but as indicated by its (appalling) name it was constructed with the specific aim of challenging strain resolution methods.

The issue of promoting the use of standardized datasets is a difficult and still largely unresolved one, as illustrated by several recently published binning methods (e.g. Wang Z et al., Luo Y et al., Yu G et al., and Xing X et al) that did not use CAMI datasets despite dealing with contig-based binning.

On the other hand, as we spent a significant amount of time a couple of years ago to build a (published and publicly available) controlled dataset simulating 50 microbiomes sampled from a pool of 700 genomes (thus matching CAMI's high-complexity dataset in terms of genomes), we decided to use the latter.

Note however that we used CAMI’s binning accuracy metrics from Meyer at al. to measure the binning performance of our algorithm on the test datasets.

4. Regarding to the methods, one of the most important parameters for any unsupervised algorithm is the determination of cluster number; however I did not see how the authors get this number or numbers and/or the stop condition such as certain thresholds. Maybe I missed it somewhere, but perhaps the authors can invest more effort in describing the methods more clearly.

This was described in the section "Initial estimate of genome richness and number of components".

For the evaluation experiments based on synthetic microbiomes of controlled complexity (e.g. the virtual cohort of 50 individuals, where each microbiome consisted in 100 genomes drawn from a pool of 750 genomes under a given abundance distribution), the number of clusters was set to roughly match the number of distinct genomes in the union of the samples.

For the analysis of real-world data (e.g. the LifeLinesDeep cohort), where the total number of genotypes is unknown, a meaningful number of clusters was estimated on the basis of the number of distinct rpS3 ribosomal protein sequences clustered at 98% identity, which roughly corresponds to species level delineations according to Sharon et al., 2013 (https://www.ncbi.nlm.nih.gov/pubmed/22936250).

5. The figures are way too small and cannot be seen without a magnifier. Please address this problem.

We improved the rendering of the figures in the present version.

6. How are precision and recall determined?

These metrics were computed as in equations 10 and 11 from Meyer et al., with precision corresponding to what the authors refer to as purity and recall corresponding to completeness.

In the evaluation experiments relying on the controlled bacterial communities, the read to genome assignments (ground truth) are known in advance for all the reads, hence the precision and recall quantities can be directly computed from the read clustering output.

Briefly, each bin is first mapped to its most abundant (in terms of number of reads) genome (note that if each bin is mapped to a single genome, a given genome can be mapped to multiple bins). Precision is then defined as the ratio of reads originating from the mapped genome to all the bin's reads. Recall on the other hand reflects how complete a bin is with respect to the sequences of its cognate (mapped) genome. Average precision is the fraction of correctly assigned reads for all assignments to a given cluster averaged over all clusters, while average completeness is averaged over all genomes (including those possibly not assigned to any cluster). We follow Meyer et al., in order to give larger bins higher weight in performance

determinations (cf the equations in the section "Comparison of read-binning algorithms").

We also used homogeneity and completeness metrics (Rosenberg & Hirschberg 2007), which provide another way to inform a cluster labeling given a ground truth (a clustering result satisfies homogeneity if all of its clusters contain only data points which are members of a single class, while a clustering result satisfies completeness if all the data points that are members of a given class are elements of the same cluster), with no sensible difference in the results.

7. Have the authors tried other k lengths? If not, why 31?

The key requirement is that k-mers should be sufficiently long so that most of them will be specific to each genome, thereby capturing genuine abundance patterns of individual genomes.

The k-mer length in our experiment (31) was chosen to be close to the value used by the authors of the LSA method to analyze their largest (terabase-sized) dataset (Cleary et al.,).

We did some limited experiments with varying k length values on smaller subsets of the data to check that small variations in k length did not result in disproportionate differences in clustering outputs.

In choosing the k length, we were also guided by the observations in Koslicki et al., that k-mer similarity between genomes at different k approximates various degrees of taxonomic similarity, and that k=31 corresponded to species-level similarity. We also noticed that k=31 is the default in the popular sequence classifier kraken.

8. I do not understand Figure 2 and its corresponding section "partial strain separation" at all. I do not think the authors are conducting pan-genome analysis in this manuscript. Then why mention it here? What does it have to do with the rest of the manuscript? What does the x-axis "cluster #" mean, and how to interpret the Figure?

The experiment conducted here was really about strain-separation (hence the wording "pangenome" that is frequently used to designate the set of strains for a given species). The pool of (real) genomes sampled to build the synthetic microbiomes (the "virtual cohort" used in the test phase) contained dozens of related strains, and we wanted to assess the behavior of the binning pipeline when facing the difficult task of separating closely related strains (up to 99.96% ANI).

The two panels of Figure 2 illustrate two practical examples of partial strain separation achieved with the method. The left panel illustrates a separation attempt of 7 strains from the Bacillus amyloliquefaciens species, while the right panel shows similar results for 8 strains of the Sulfolobus islandicus species.

As the genomic origin of each read is known in the virtual cohort dataset, the figure shows for each strain (represented by a horizontal line), the distribution of its reads among the full set of clusters/bins generated by the pipeline, and arbitrarily ordered along the x-axis (cluster # stands for cluster number).

The left panel illustrates that the 7 strains from the B. amyloliquefaciens species are mostly separated into two groups according to whether their "core" cluster corresponds to bin ~220 or bin ~500.

The right panel on the other hand shows that the 8 strains of the S. islandicus species share a common "core" cluster (located near the x origin), while variable portion of their genomes are mapped to distinct "variable" clusters.

We wanted to mention these results in the manuscript because we feel that they embody an important limitation of the method, as the strain separation problem is likely to arise in any joint analysis of a sufficiently large number of related samples that are likely to contain distinct but closely related organisms.

9. From Table 2 I can probably fathom that the binning accuracy is good because the algorithm attempts to cluster everything into as many cluster as possible in order to avoid contamination. As a result the algorithm achieves very good contamination-free level but creates massive amount of partial genomes. Indeed The authors have discussed the "inevitability" of this problem in Discussion, but I still suggest the authors to make more concrete suggestions also in Discussion such that people can make continual improvements based on this work.

We should first clarify that the low contamination figures in Table 3 (former Table 2)are not the result of some sort of internal tuning of the algorithm aiming to specifically optimize this criterion, and that the completeness and contamination metrics were only measured post-binning.

We should also mention that the production of many pure partitions is balanced by the concomitant generation of large and unresolved partitions.

The production of these unresolved partitions (whose counts are now also included in a new version of the table as the previous version only focused on the completeness of low contamination partitions) appears unavoidable (hence the "inevitability" of the problem in the discussion) because ultimately linked to the fact that the extent of genome divergence is not uniform across the range of taxa occurring in the samples.

As discussed above, strain-level ("pangenomic") variation is another factor contributing to cluster fragmentation, resulting in differential segregation of core and variable portions of genomes. As pointed by the reviewer, this limitation is rather inherent to the read aggregation across samples, and there are no clear workaround to deal with it (e.g. a form of "soft-clustering" allowing "core" sequences to belong to more than one "variable" cluster could improve genome completion statistics but does not appear very satisfying to us).

10. Perhaps the authors can compare their reads clustering algorithm with other reads clustering algorithms?

We did that experiment when comparing our algorithm against the state of the art read-binning method LSA (Table 1).

It could be interesting to note that, even though the dominating paradigm nowadays is assembly-first binning, the very first binning methods reported, like AbundanceBin and MetaCluster, operated at the read level. This shift towards contig binning today was probably mainly driven by the increase in data throughput, as the first read-level binning methods were designed at the time of 454 and even Sanger sequencing (both providing longer reads) and to operate on individual samples. They were thus not designed to scale to large multisample short read datasets like the presented method. In that perspective, assembly acts as a pre-processor to reduce the computational burden of binning.

Besides the two pioneering read binning methods mentioned (AbundanceBin and MetaCluster), we are aware of CompostBin (Ref), which is a PCA-based read level binning algorithm, but that was designed and only tested on Sanger reads.

BiMeta and MetaProb operate at the read-level but describe themselves as "assembly-assisted" (resting on the detection of read overlaps). BiMeta was tested on 454 reads simulating bacterial communities of a dozen of different genomes at most, and on the Acid Mine Drainage real-world dataset, which is of low complexity and consists in Sanger reads.

MetaProb shares some principles with BiMeta; it is also "assembly-assisted" and was tested on the same low-complexity synthetic datasets as the latter. They also tested their method on a real microbiome sample consisting in 43 million reads, but only after filtering the latter down to 2 million reads.

Thus, all these methods were designed to operate on a individual samples, at a time were scalability issues were less acute than today.

On the other hand, besides AbundanceBin that makes use of a coverage signal derived from long (unique) k-mers, the other methods are better described as composition-based, using a nucleotide composition signal measured from short k-mers (typically of length 4 or 5).

We developed our method with scalability in mind, as we wanted it to be able to process on the order of 10^10 short reads and to be able to process increasingly larger multi-sample datasets by simply adding additional computing resource.

In this respect, there is only one competing method remaining, LSA (Cleary et al.,), the read-binning method we refer to as the state of the art, and against which we demonstrated the benefits of recasting the original read-binning problem into the sparse coding framework.

Reviewer #2: The article "Binning unassembled short reads based on k-mer covariance using sparse coding" by Kyrgyzov et al., describes a new method for binning extremely large datasets on read-level to obtain MAGs.

First, I highly welcome new approaches that tries to tackle the MAG recovery by binning prior to assembly instead of post-assembly as most studies currently do. Secondly, I am no code-expert. Hence, the new approach seems impressive but I can not evaluate the merrit compared to the previous efforts in this regard.

Instead I have focused my review from a user's perspective and several reservations that needs to be addressed and clarified. However, my main concern is related to what the authors state in the discussion "As both the sensitivity and resolution of covariance-based methods increase with the number of samples, it is reasonable to anticipate further gains in the application of such methods in relation to future increases in the scale of sequence data generated (e.g. increased cohort sizes)." I must say that with only 14 nearly complete genomes from 1135 samples and 10 Tbp of data this is not scaleable. In this case culturing or single cell sequencing seems much preferable.

We will try to clarify and address sequentially the different items raised by the reviewer in this comment.

i) About our statement ***"As both the sensitivity and resolution of covariance-based methods increase with the number of samples, ..."***

This statement simply underlines a property of measuring abundance co-variation profiles across many samples: the number of distinct profiles grows exponentially with the number of samples; hence the larger the number of samples, the larger the number of distinct possible profiles and the (theoretical) possibility to distinguish the genomes from each other.

ii) Regarding the use of the term "scalable/scalability"

By scalability, we have in mind the technical meaning of the term, which refers to the ability of an algorithm to adapt to order of magnitude change in the input by simply providing additional computer resources, and its ability to maintain its functionality and performance under high demand (i.e increasingly higher data volumes). Practically, we wanted the method to be able to deal with multi-terabases datasets like the LLDeep cohort (> 10^10 reads).

iii) About the fact that the number of complete genomes is low in comparison of what can be recovered through assembly-based approaches.

We can only agree with that point, and this is already apparent by the additional smaller-scale experiment we performed to compare assembly-first versus bin-first approaches.

However, we would like to first restate that our aim was not to surpass assembly-first methods were they excel most (i.e. recovering high abundance genomes), and that our focus was instead on the recovery of lower abundance genomes. In that respect, even though the number of complete genomes is low, we think the main value of the method lies in the hundreds of pure but incomplete partitions/bins generated, as the majority of these appear new (i.e. yield no matches against reference genome compendia).

On the other hand, we acknowledge several important limitations to the method, which we hope will spark some interest ultimately leading to incremental improvements of the method.

In particular, the second additional experiment we performed sheds some light on a key issue: the fact that only 3 out of 7

complete genomes recovered from the analysis of a subset of 18 samples were also present among the set of MAGs identified in the analysis of the full cohort (1135 individuals) is indicative of a lack of stability of the method, likely related to the occurrence of more extensive strain variation in the full dataset that leads to fragmentation of these genomes into smaller incomplete (though pure) partitions.

On the positive side, we have shown (including in a new specifically designed experiment) that the genome sets recovered by both strategies show limited overlap (even though the assembly-based approach yields significantly more complete genomes), thus supporting the complementarity nature of the two approaches.

It should also be noted that the samples from the LLDeep cohort were characterized at relatively low sequencing depth, compared to the study of Pasolli mentioned by the reviewer which generated roughly 2 times more sequence data per sample (5.3 Gb average per sample *after* quality control in Pasolli et al., versus 3.0 Gb data *before* quality control in LLDeep).

Last but not least, assembly-first approaches are inherently unable to recover genomes consistently segregating at low coverage, while bin-first methods are able to enrich such reads into assemblable coverage, as shown in the first additional experiment performed here and on the real-life data by characterizing (across all the samples) the relative abundance of reads enriched in selected MAGs (section "recovery of low-abundnace genomes" and Figure 4).

General comments:

- Most current studies use a single-sample assembly approach to limit strain-diversity and hence increase the chance of assembly and the risk of strain-contaminatied MAGS (see e.g. Pasolli et al., 2019). This study does the exact opposite and tries to maximize the strain-diversity across samples. Hence, without some sort of strategy to counter this I do not see how this will become useful. One strategy could be to "sample-optimize" assembly by using the samples with the least strain-diversity that reach a given coverage to maximize the chance of genome recovery.

We need first to clarify that our method does not seek "to maximize strain-diversity across samples", although we acknowledge, and make clear in the manuscript, that it is vulnerable to strain-level variation. We agree that sample by sample assembly limits the risk of strain mix-up, but at the

expense of focusing on those genomes that reach high-coverage (around 10x).

Our approach aimed at relaxing the latter constraint, but by doing so through the aggregation of lower abundance reads across samples, it becomes indeed vulnerable to strain-level variation.

It should be noted that, to some extent, the degree of similarity that one wishes to distinguish can be tuned through the choice of a k-mer length. Increasing the latter would increase the separation of closely related sequences, but only to some extent because the locality sensitive hashing scheme will by nature increase the probability of collision for similar sequences.

This property leads to a benefit/disadvantage balance: the benefit is that this allows to conveniently deal with sequencing errors (noise), the disadvantage is that this also inherently limits the power to separate very similar sequences (e.g. strains).

We probably underestimated the extent of strain-level variation in real-world data, and the high-level of genome fragmentation in the LLDeep partitions can be partly attributed to this problem (low sequence coverage being another driver).

To the best of our knowledge, a method that could target, in an unsupervised way, low-coverage genomes in a strain resolved way is not available today.

- With the current strategy for recovering low-abundant populations I think it would be important to mention culturing and single-cell sequencing. I think this method is "competing" with those and not really "normal" MAG recovery.

Thank you very much for this insightful remark. Indeed, we think our work could be better understood that way, and we tried to change the formulation of the scope of the manuscript accordingly.

- The main reason why read-binning is not used is due to the relatively extreme variance that can be locally in genome composition and coverage. E.x. Local sequence variation in GC content which amplifies method biases and stocastic variation which is very high at low coverage. Hence, without any assembly-graph to get stable long-range coverage estimations I think it would be difficult for the authors to come close to binning complete genomes by starting from the reads. If decent quality

MAGs are the aim I would hope that the authors would spend their effort in binning at the assembly-graph stage instead.

Even though we agree with parts of this insightful remark, we believe it should be mainly directed at nucleotide composition-based binning methods, that rely on relatively short k-mers (4 or 5 mers) and for which intra-genome signal variations are an important issue, especially for GC-poor genomes.

Even though stochasticity could impact signal/noise ratio for low abundance genomes in individual samples, we expect this to be counterbalanced by the increased strength of the covariation signal resulting from the processing of larger number of samples.

On the other hand, there were several attempts to cast binning as an assembly-graph partitioning problem (see for example Pell et al., PNAS), but as far as we are aware of, this has not yet been widely adopted. We believe however that this represents another valuable ongoing venue of research, e.g. https://www.biorxiv.org/content/10.1101/462788v2.full

- If you want to compare with MAGs then I suggest to use the dataset from Pasolli et al. which will enable direct comparison with state-of-the-art single-sample assembly based approaches.

The scope of the impressive study of Pasolli et al appears much wider than ours, both in terms of the number of samples analyzed (10,000) and the biological questions investigated, while our paper has a much narrower and technical scope.

Having said that, we agree it would have been valuable to perform both assembly-first and bin-first approaches on this dataset, but this appears practically out of reach given the sheer amount of time and resources that would be required to carry out such a comparison.

One should also keep in mind that we started our work (in 2017), well before the Pasolli analysis got published (early 2019), and that our human resources were more limited as well (a postdoc researcher and (partly) a PhD student).

- The discussion lacks direct comparisons with real data and datasets from other studies. E.g. several times it is mentioned that "normal" assembly based approaches have problems with strains (which is definitely true). However, in my mind single-sample assembly approaches should be much superior to the approach in this article which maximizes strain-diversity.

Hence, I much more direct comparison is needed with state-of-the-art approaches. E.g. Pasolli et al., 2019 (but there is a quite large number of papers with similar approaches).

We addressed the general request (also formulated by the first reviewer) to perform a more direct comparison of "normal" (assembly-first) versus bin-first approaches, and described and discussed the main results above and in the revised manuscript.

Briefly, the limited overlap observed between genome sets recovered by both approaches suggests they should be viewed as complementary rather than antagonistic.

We also more thoroughly discuss the impact of strain-level variation, and try to formulate the view of a trade-off between advantages and weaknesses of the method: on one hand, aggregating a coverage signal across a large number of samples enables to target low-abundance genomes, on the other hand the occurrence of strain-level variation across the samples leads to increased fragmentation and poorer genome-completion statistics.

- The discussion mentions the ability to recover low-abundant MAGs. However, in the current manuscript I did not see any of these informations? They are essential to the paper as it is what the aurthors claim make this method usefull. However, as I opened this review, I think 14 near complete genomes from 1135 samples and 10 Tbp of data is simply not nearly good enough to be usefull. There should be >>10,000 strains. Hence, it is simply not good enough to be usefull unless the authors have an angle I've missed in my review.

The information about the ability of the pipeline to recover low abundance genomes from a real-life dataset (microbiomes of the LifeLinesDeep cohort) was presented in the (initially quite small) section "Evaluation of read enrichment levels".

We also already stated that the aim of the present method was not to surpass assembly-based approaches, as we were more focused on the retrieval of rarer genomes (or portions thereof).

To assess whether we could indeed identify such consistently low abundance genomes in real-life datasets, we characterized the abundance of a dozen of completely reconstructed genomes from the LifeLinesDeep cohort analysis by directly mapping the raw reads of the original samples against them. Given the large size of the cohort, this analysis was not performed on the full set of MAGs but on a limited number of genomes, the aim being to validate the ability of the method to retrieve such genomes.

Figure 4 illustrates some results from this analysis, with the left panel showing an example of a consistently low-abundance genome (i.e. with nearly all the samples contributing 10^-5 to 10^-4 of their reads to the given genome), while the right panel shows a genome of overall moderate abundance (10^-4) but reaching higher abundance (10^-3) in a few dozens of samples (represented by the rightmost peak in the histogram).

We made this result more apparent in the new version of the manuscript, as it represents an important feature and/or appeal of our method.

In addition, one of our complementary experiments illustrates the archetypal case of a given genome segregating consistently at low abundance across an entire set of samples. As a matter of fact, this genome would not reach, in any single sample, the coverage required to yield kilobase-sized fragments by assembly.

- Section "Enhaced binning accuracy": Please briefly sentence describe the dataset. How many genomes and in which abundance patterns. Furthermore, the "presions, recall and F-value" should be split on a genome-basis. There might be a large genome-specific component?

Beyond simply pointing to the original publication, we have now provided a more complete description of this dataset in the new version of the manuscript. Briefly, this dataset consists in 50 microbiomes containing 100 distinct genomes each, sampled from a reservoir of 750 completely sequenced genomes under a power-law distribution of parameter 1 and a mean coverage of 10x.

Figures and tables:

- Figure 1: Convert to a table. It's currently very hard to read. Describe what sparse coding (this study?) is in the legend.

Done.

- Figure 2: The quality in my version of the downloaded pdf is almost unreadable. I also have a hard time to understand the figure. I assume it was a "mock" spike in to a complex sample. Hence, it should be possible to show the both actual number of reads binned and percentage of the theoretical strain-specific recovery. Finally, how similar were the strains and in which abundance were they spiked in the samples and how many samples were used for binning?

Your understanding of the figure is absolutely correct: these results were generated during the test experiments that involved

a virtual cohort of 50 microbiomes containing 100 distinct genomes (including strain-level variation) each.

We provide now a complete description of the set of strains (8 B. amyloliquefaciens strains for the left figure, 7 S. islandicus strains for the right figure) involved in these experiments, with strain similarities (ANI) being presented in a separate table.

- Tabel 1: Please use the accepted standards for MAG quality and remember the rRNA/tRNA criteria for HQ MAGS which is easily missed (Bowers et al., 2017). How many of these could be considered low abundant (i.e. in < 10 x coverage in a single sample) this is essential information for the analysis and referred to in the text. However, currently no data is available (I might have missed a supplementary?)? CheckM can in cases with strain-diversity overestimate the completeness as the core-genome might co-bin and assemble due to high similarity. Please use some of the exsisting tools for an estimation of MAG strain-diversity e.g. CMseq from Pasolli et al., 2019.

Some elements to deal with this comment have already been discussed above. Basically, a systematic labelling of the MAGs as low abundance if < 10x in individual samples, as suggested by the reviewer, would be computationally prohibitive, as this would entail either assembling or mapping all the reads from all the samples (n=1135) against the MAGs. This is the very reason we performed the read enrichment analysis (involving the mapping of all the reads of all the samples) on a subset of MAGs. On one hand, this made the relative abundance estimate tractable. On the other hand, the main objective of this analysis was to demonstrate that the method could indeed recover low abundance genomes (like the genome shown on the left panel of figure 4). In that respect, our study is probably best viewed as a proof-of-concept study.

Regarding the bin quality metrics, we sticked to checkM because, even though these are perfectible, they represent the gold standard of bin quality checking, e.g. the quality assessment of the "Metagenome Bins" in DoE's Integrated Microbial Genomes & Microbiomes (IMG/M) system relies exclusively on checkm's completeness and contamination metrics, and it is used for this purpose by numerous studies, for example Muriovec et al., for a very recent work.

- Figure 3: Quality in my version of the downloaded pdf is almost unreadable.

We have improved that in the present version.

- Figure 5: Add labels to each axis. Adjust the gc axis to a relevant range. It does not make sense to have a negative coverage axis. What is the color? a+c) The large variations in coverage indicate potential large problems with strain variation in the bin. Add the the checkM and CMseq values for these bins on the figures. b+d) The figure is hard to read. What is the read-abundance on the x-axis? Make it a "relevant" coverage scale instead of number of reads.

We put labels, adjusted axis and added the checkM completion and contamination values in the legend.

The right panels are histograms of the same type as those shown in Figure4: they display the number of samples (on the y-axis) that contribute the fraction of their reads shown on the x-axis to the genome that is depicted (and whose gc-coverage plot is shown on the corresponding left panel).

References:

All references pointed to in the above text are included in the manuscript, with the exception of:

Wang Z, Wang Z, Lu YY, Sun F, Zhu S. SolidBin: Improving Metagenome Binning with Semi-supervised Normalized Cut. Bioinformatics. 2019 Apr 12.

Luo Y, Yu YW, Zeng J, Berger B, Peng J. Metagenomic binning through low-density hashing. Bioinformatics. 2019 Jan 15;35(2):219-226.

Yu G, Jiang Y, Wang J, Zhang H, Luo H. BMC3C: binning metagenomics contigs using codon usage, sequence composition and read coverage. Bioinformatics. 2018 Dec 15;34(24):4172-4179.

Xing X, Liu JS, Zhong W. MetaGen: reference-free learning with multiple metagenomic samples. Genome Biol. 2017 Oct 3;18(1):187.

Murovec B, Deutsch L, Stres B. Metagenome-Assembled Genomes Orchestra (MAGO): computational framework for high-quality production and large-scale evolutionary analysis of metagenome assembled genomes. Mol Biol Evol. 2019 Oct 21.

Manuscript Click here to download Personal Cover Cover_Letter.pdf

Dear Dr. Zauner,

First of all, we would like to thank you and both reviewers for the valuable and useful feedback.

As per your suggestion, we hereby resubmit a significantly recrafted version of our manuscript that includes additional material and takes the remarks of the two reviewers into account.

Three major revisions shape the new manuscript:

1- Several comments from the reviewers made us realize that the presentation of our read binning method was perceived as competing with assembly-based binning, which is definitely not the message we intended to convey. Instead, our aim was to illustrate the relevance of our method for targeting lower abundance genomes, which indeed can fail to be recovered by assembly-first approaches but by no means should imply that it surpasses assembly-based approaches in the domain where they excel most (i.e. recovery of abundant genomes) or in terms of overall genome recovery yields. As noticed by the second reviewer, our method could be better viewed as sharing the motivation of single-cell techniques, by "in silico" targeting rarer genomes. We tried to clarify this misunderstanding in the present version, and were greatly helped in this effort by two additional experiments suggested by the reviewers and whose results we summarize in the next two points.

2- We present and discuss a small experiment performed in order to demonstrate the ability of a bin-first protocol to recover a low abundance target genome that fails (almost by construction) to be recovered by assembly-first binning. We also extended the section about the recovery of low abundance genomes from the real-life microbiomes in order to emphasize this aspect of the analysis.

3- We performed a direct comparison of assembly-first versus bin-first approaches in terms of the number of complete genomes retrievable by each. This experiment sheds light on a few key issues that are more extensively developed in the new manuscript. Most prominent among these is the observation of limited overlap between the genome sets reconstructed by both strategies -even though the assembly-based approach yields significantly more complete genomes-, thus supporting the complementarity nature of the two strategies.

We hope this new manuscript will address most of the concerns raised by the reviewers and increase its relevance to a larger audience, while preserving our initial aim which was to present a new methodology improving over a state of the art read-level

binning tool (namely the LSA algorithm, Table1), without concealing important limitations that we hope will raise the interest of the community so as to lead to continual improvements in this underexplored (compared to mainstream assembly-first binning) research direction.

Yours sincerely,
Thomas Brüls
