# Supplementary material for: Binning unassembled short reads based on k-mer abundance covariance using sparse coding
Source: Gigascience. 2020 Mar 29;9(4):giaa028. doi: 10.1093/gigascience/giaa028 (PMC7099633; doi:10.1093/gigascience/giaa028)
Supplement: giaa028_Supplemental_Files [file giaa028_supplemental_files.zip › Supplementary_Table1.pdf]

cluster id	# of spiked reads	# of reads	ratio
0	2782	5106	0.544849
1	0	6164	0.000000
2	0	7284	0.000000
3	0	4352	0.000000
4	0	5774	0.000000
5	6	6594	0.000910
6	0	4498	0.000000
7	0	5134	0.000000
8	0	6380	0.000000
9	0	7206	0.000000
10	0	5490	0.000000
11	0	6220	0.000000
12	0	6574	0.000000
13	0	6252	0.000000
14	0	6230	0.000000
15	0	6624	0.000000
16	0	5600	0.000000
17	8	6704	0.001193
18	2	6802	0.000294
19	2	67812	0.000029

Table 1: Cluster assignments of reads from a target genome versus background (unrelated) reads. Nearly all the 2800 reads from the target genome segregating at low levels in the samples (100 paired-reads per sample in 14 samples, none in the remaining samples) are binned in a single partition using our bin-first pipeline, leading to the complete genome after assembly. No kilobase-sized contig could be assembled from any individual sample, making the assembly-first protocol inoperable (see main text).
