# Supplementary material for: Binning unassembled short reads based on k-mer abundance covariance using sparse coding
Source: Gigascience. 2020 Mar 29;9(4):giaa028. doi: 10.1093/gigascience/giaa028 (PMC7099633; doi:10.1093/gigascience/giaa028)
Supplement: giaa028_Response_to_Reviewer_Comments_Original_Submission [file giaa028_response_to_reviewer_comments_original_submission.pdf]

Author's Response To Reviewer Comments

Close

Dear Hans,

We would like to thank you and the reviewer(s) for your work and the positive evaluation. We have now made the requested corrections to our manuscript (Kyrgyzov_R2) and updated our GigaDB repository.

With best wishes,
Thomas

Close
